# Oceanographic drivers of the vertical distribution of a highly migratory, endothermic shark

**DOI:** 10.1038/s41598-017-11059-6

**Published:** 2017-09-05

**Authors:** Daniel M. Coffey, Aaron B. Carlisle, Elliott L. Hazen, Barbara A. Block

**Affiliations:** 1 Tuna Research and Conservation Center, Monterey Bay Aquarium, 886 Cannery Row, Monterey, CA 93940 USA; 2 Hopkins Marine Station, Stanford University, 120 Oceanview Boulevard, Pacific Grove, CA 93950 USA; 3 Environmental Research Division, Southwest Fisheries Science Center, National Oceanic and Atmospheric Administration, 99 Pacific Street, Suite 255A, Monterey, CA 93940 USA; 4 Present Address: Hawaii Institute of Marine Biology, University of Hawaii at Manoa, PO Box 1346, Kaneohe, HI 96744 USA

## Abstract

Salmon sharks *Lamna ditropis* are highly migratory, upper trophic level predators in North Pacific ecosystems. We analysed a multi-year satellite tag dataset to investigate the habitat use of female salmon sharks across their broad range in the eastern North Pacific (NEP) and identified key environmental factors that influence vertical distribution. Salmon sharks displayed remarkable plasticity in habitat use across disparate oceanographic regions in the NEP and increased utilization of deeper waters in offshore habitats. Diel shifts in vertical distribution and behaviour were consistently observed across their range and likely reflect shifts in their foraging ecology. Salmon sharks utilized a broad thermal niche and exhibited submergence behaviour, possibly for thermal refuge, when encountering sea surface temperatures outside their preferred temperature distribution. Moreover, the vertical distribution of salmon sharks indicates they were able to exploit low dissolved oxygen environments (<1–3 ml l^−1^), occasionally for extended periods of time in offshore habitats. However, salmon sharks generally reduced their use of deeper waters when encountering the combination of cold temperatures (<6 °C) and low dissolved oxygen concentrations (<1–3 ml l^−1^). Combining vertical distribution with high-resolution horizontal movements furthers our understanding of the ecological and environmental drivers of movement across short (diel) and long-term (migratory) scales.

## Introduction

Investigating patterns of habitat use and movements of top predators is fundamental to understanding their ecology and plays an important role in fostering effective management strategies^1–3^. As highly migratory, upper trophic level predators in North Pacific ecosystems, salmon sharks *Lamna ditropis* likely play an important ecological role across a range of neritic and pelagic habitats^4–8^. In order to elucidate the ecosystem role of these pelagic predators across their broad distribution it is important to describe their habitat use across their range and investigate how their vertical distribution is influenced by changes in biological and environmental conditions.

Previous satellite tagging studies of salmon sharks in the eastern North Pacific (NEP) have only included female sharks due to the dramatic sexual segregation that occurs in the North Pacific^9^, and have revealed that they are highly migratory throughout the California Current, Alaskan Gyre, and Subtropical Gyre^1, 6–8^. During summer (June 21–September 21) and autumn (September 22–December 20) months, female salmon sharks aggregate in the productive, subarctic waters of the Gulf of Alaska and undergo a seasonal migration in the winter (December 21–March 19) with some individuals migrating to temperate and subtropical regions^6–8^. However, some female sharks remain and overwinter in subarctic waters^6–8, 10^. In spring (March 20–June 20), the migrating sharks’ habitat extends into warmer, oligotrophic waters in the Subtropical Gyre as well as the productive waters of the California Current^7, 8^. Electronic tagging has revealed that salmon sharks utilize a broad vertical and thermal niche, exceeding depths of 1,800 m and occurring at temperatures ranging from 2 to 24 °C across their distribution^7, 10^. This extensive geographic and vertical range of salmon sharks, particularly their use of cold high latitude waters, is likely associated with their high degree of endothermy and unique cardiac physiology^7, 11–13^.

While horizontal migratory patterns of female salmon sharks in the NEP have been well-studied^1, 6–8^ much less is known about how their vertical distribution varies across their expansive range. Seasonal changes in the depth distribution of salmon sharks have been relatively well-described in the neritic waters off Alaska, likely reflecting changes in their foraging ecology^6, 10^. Weng *et al*.^7^ described a shift in the vertical distribution of a tagged salmon shark as it migrated to the Subtropical Gyre, where it spent more time at greater depths, avoiding the surface waters, in the warmer, southern extent of its range. This is the only study to describe the vertical distribution of salmon sharks in pelagic habitats, yet it was based on four individuals and did not encompass their full geographic distribution.

Because patterns in the vertical distribution of predators have direct implications for their trophic ecology and ecosystem role^14, 15^, overlap with commercially important species^10, 16^, and vulnerability to fishing pressure^17^, a more comprehensive characterization of vertical distribution across the full range of salmon sharks is needed to further elucidate how these top predators are exploiting the neritic, epipelagic and mesopelagic habitats of the NEP and foster improved management practices. In addition, examination of vertical distribution in relation to environmental data, such as temperature and dissolved oxygen (DO), will enhance our understanding of the oceanographic drivers of these patterns and physiological capabilities and limitations of salmon sharks. We analysed an extensive satellite tag dataset to investigate the habitat use of female salmon sharks across their migratory range and identify environmental factors that may influence vertical distribution throughout the major ecoregions of the NEP.

## Results

### Data recovery

The mean deployment length (±s.d) for the 65 pop-up satellite archival tags (PAT) analysed, including 11 recovered archival records, was 178 ± 89 days (11,578 total days) spanning 2002–2008. Incorporating 24-h archival data (mean deployment 129 ± 100 days; 1,423 total days) with 24-h and aggregated 24-h transmitted data sets resulted in 4,152 days of time-at-depth (TAD) histograms from 59 tags, 3,877 days of time-at-temperature (TAT) histograms from 58 tags, 4,209 days of profile of depth and temperature (PDT) data from 64 tags, 6,532 days of minimum and maximum depth from 64 tags, and 6,860 days of sea surface temperatures (SST) from 65 tags (Supplementary Table S1). The number of tags and days differs between datasets due to variability in success of transmission to the Argos satellite system following tag pop-up. Together these data provide extensive information for examining how salmon sharks use the oceanographic habitats of the NEP.

### Geographic distributions of tagged sharks

**Figure 1:**
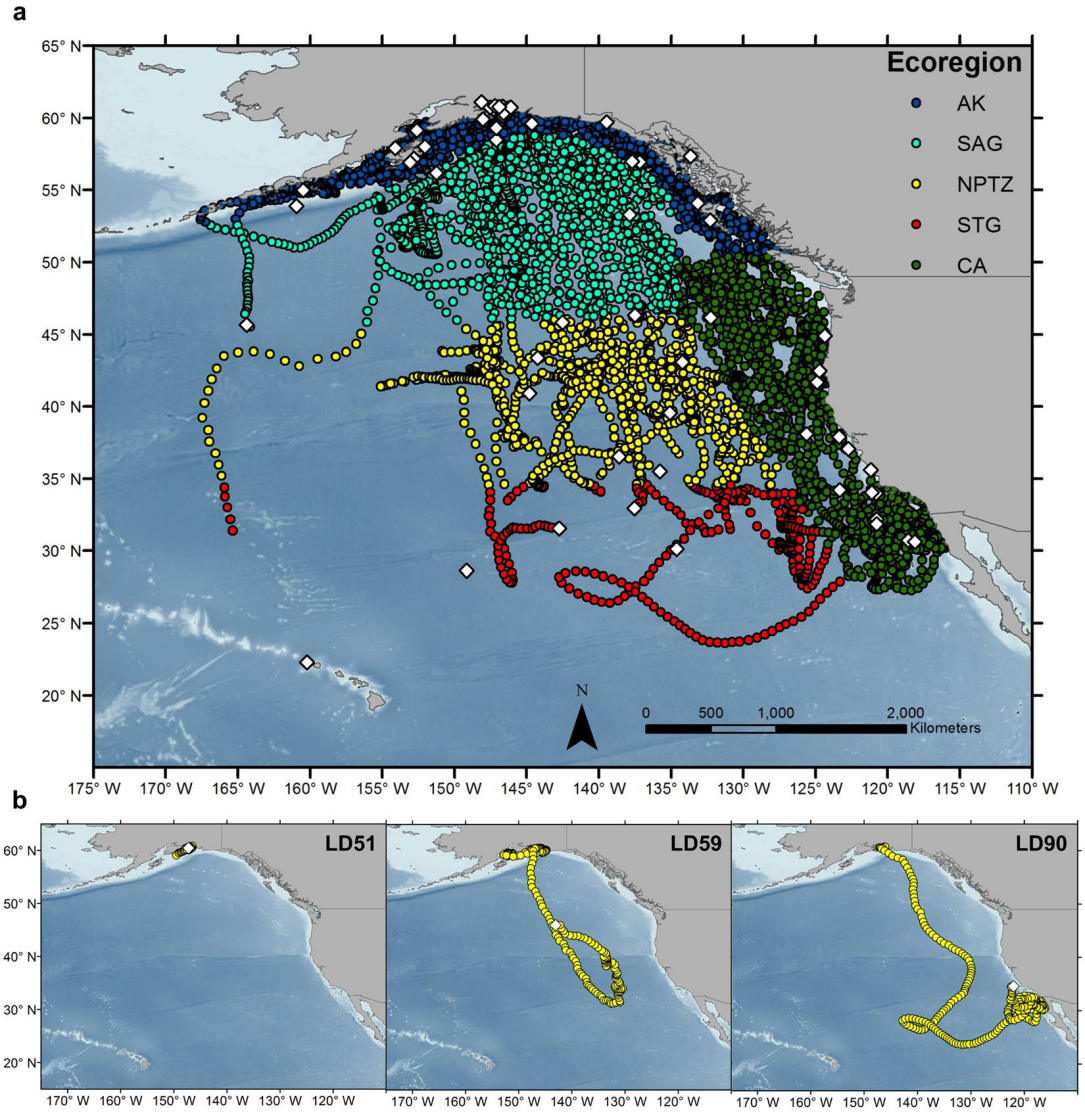
Distribution of tagged salmon sharks in the eastern North Pacific (NEP). (**a**) Positions of tagged sharks that provided usable data (n = 65) coloured by major ecoregions of the NEP: Alaska Coastal Downwelling (blue), Subarctic Gyre (cyan), North Pacific Transition Zone (yellow), Subtropical Gyre (red) and California Current (green). (**b**) Individual tracks of three sharks for which PAT tags were recovered (LD51, LD59, LD90). Circles indicate daily mean Bayesian state-space model position estimates and white diamonds indicate pop-up locations of PAT tags. Only positions that overlap temporally with PAT records are shown. Map was created using ArcGIS 10.2 (ESRI Inc., Redland, CA, USA, http://desktop.arcgis.com/en/).

Tagged salmon sharks were highly migratory and moved throughout the waters of Alaska and the NEP (Fig. 1). Electronic tagging revealed that sharks were present in AK year-round with a peak occurrence during summer and autumn months (August - December). Some sharks began migrating to lower latitudes during the autumn and winter months (September - February), with individuals spending considerable time in southern ecoregions of the NEP, in particular the California Current. LD12 migrated as far south as the Hawaiian archipelago^7^; though, there was a 64-day gap in the track between the last Argos position (31°N) and the PAT pop-up location (22°N). Tracks of sharks for which PAT tags were recovered remained primarily in the neritic waters of AK during the duration of the PAT tag deployments, although some sharks migrated to the STG and CA (Supplementary Fig. S1). LD59 migrated to the STG, spending the majority of its time offshore in the NPTZ, while LD90 was the only archival record to migrate through each ecoregion and spent a considerable amount of time in CA waters (Fig. 1; Supplementary Fig. S1).

### Vertical and thermal habitat utilization

**Figure 2:**
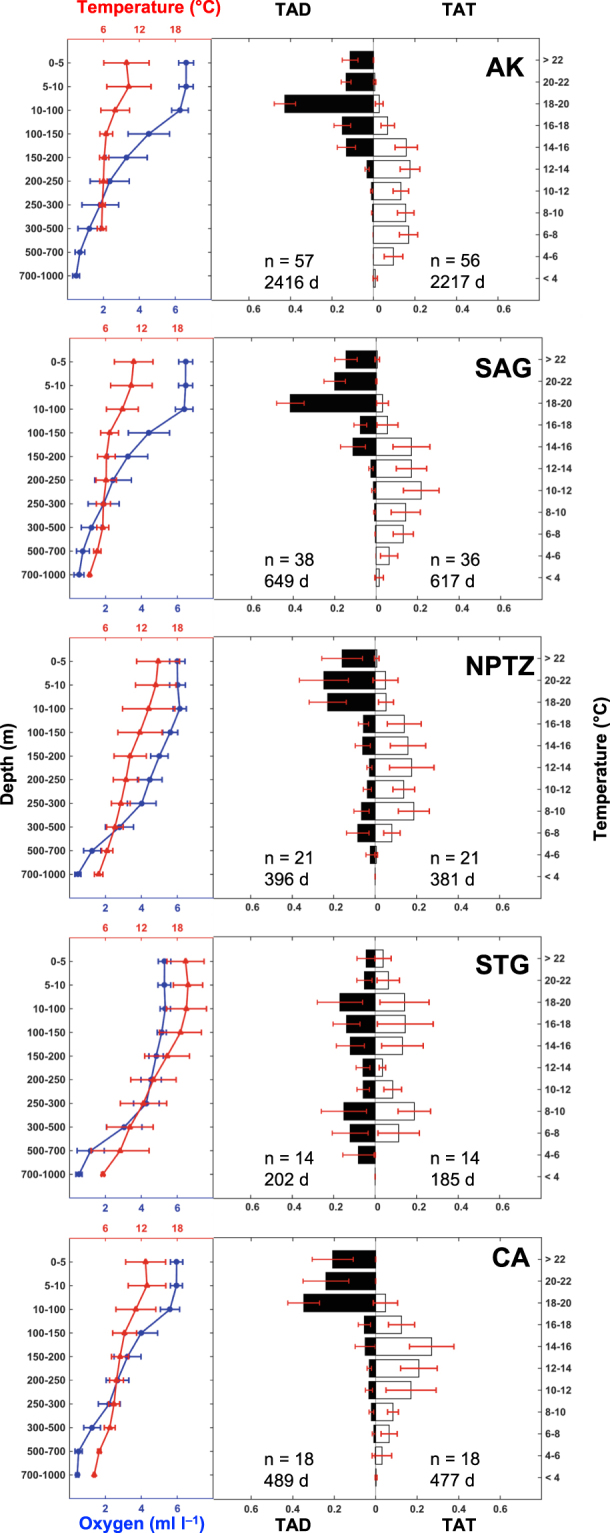
Mean proportion of time spent at depth (TAD) and temperature (TAT) for all sharks aggregated by ecoregion. Histogram error bars represent 95% confidence limits. Total number of sharks (n) and days of histogram data (d) are shown according to each ecoregion and dataset. Mean temperature profiles (red) were constructed from profiles of depth and temperature (PDT) data from the PAT tags. Mean dissolved oxygen profiles (blue) were constructed from monthly data obtained from the World Ocean Atlas 2013 along the track of each shark. Profiles were aggregated by ecoregion across each programmed depth bin. Profile error bars represent ± one standard deviation (s.d).

The depth and temperature distribution of sharks changed consistently when migrating between the different ecoregions of the NEP (Fig. 2). In AK waters, salmon sharks spent the majority of their time between the surface and 200 m (95%), with a peak depth use between 10–100 m (43%) and regularly encountered temperatures between 4–16 °C (89%). However, given that data for sharks in AK spanned all seasons, these patterns represent an annual integration of seasonal changes in their vertical and thermal habitat (see Carlisle *et al*.^10^). Sharks spent more time at shallow depths (76% in top 100 m, 35% in top 10 m) in the waters of the SAG and experienced similar temperatures (83%, 6–16 °C) as AK. As sharks migrated to the NPTZ they increased their use of deeper waters (64% in top 100 m, 15% between 300–700 m) and had a warmer temperature distribution (80% in 8–18 °C) compared to subarctic waters. In contrast, sharks in the STG spent the least amount of time at shallow depths while exhibiting a deeper bimodal distribution (43% between 10–200 m, 27% between 300–700 m) compared to the NPTZ. TAT data reflected this bimodal distribution (42% between 14–20 °C, 30% between 6–10 °C; Fig. 2). LD12 consistently remained deeper than 100 m and exhibited a deeper bimodal distribution (36% between 150–250 m, 46% between 500–100 m; maximum depth 832 m) and had a warmer temperature distribution (29% between 20 to >22 °C, 34% between 8–12 °C) when migrating south from 31°N towards the Hawaiian archipelago. Similar to the SAG, sharks in CA primarily inhabited waters between the surface and 100 m (80%); however, they had a warmer temperature distribution (77% between 10–18 °C) and spent the most time in the top 10 m of the water column (45%) compared to other ecoregions.

### Time spent at different oxygen concentrations

**Figure 3:**
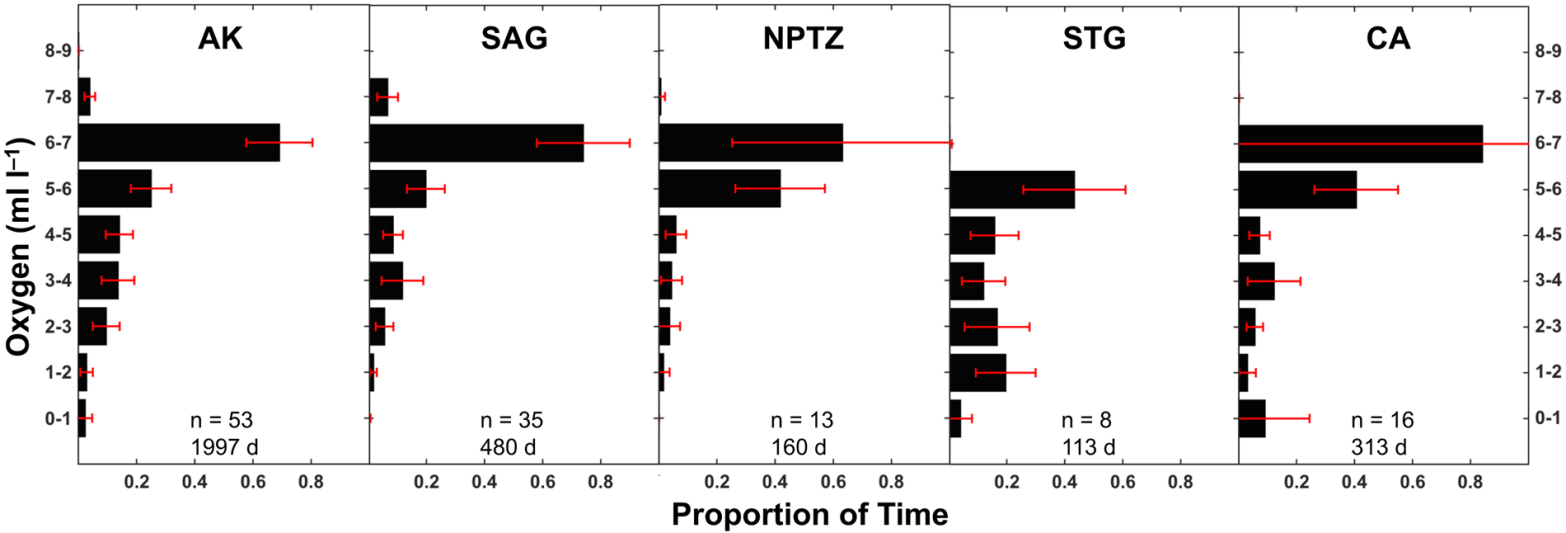
Estimated mean proportion of time spent at oxygen (TAO) for all sharks aggregated by ecoregion. Total number of sharks (n) and days of histogram data (d) are shown according to each ecoregion. Error bars represent 95% confidence limits.

Estimated time spent at different oxygen concentrations (TAO) revealed tagged salmon sharks spent the majority of time at DO concentrations ≥6 ml l^−1^ (64–84%) across all ecoregions, with the exception of the STG, which is characterized by lower surface mixed layer DO concentrations, where sharks most often experienced DO concentrations between 5–6 ml l^−1^ (43%; Fig. 3). Salmon sharks utilized waters with low DO concentrations (<1–3 ml l^−1^) in all ecoregions; though, overall sharks spent the most amount of time at low DO concentrations in the STG (40%) and CA (18%).

### Diel behaviour

**Figure 4:**
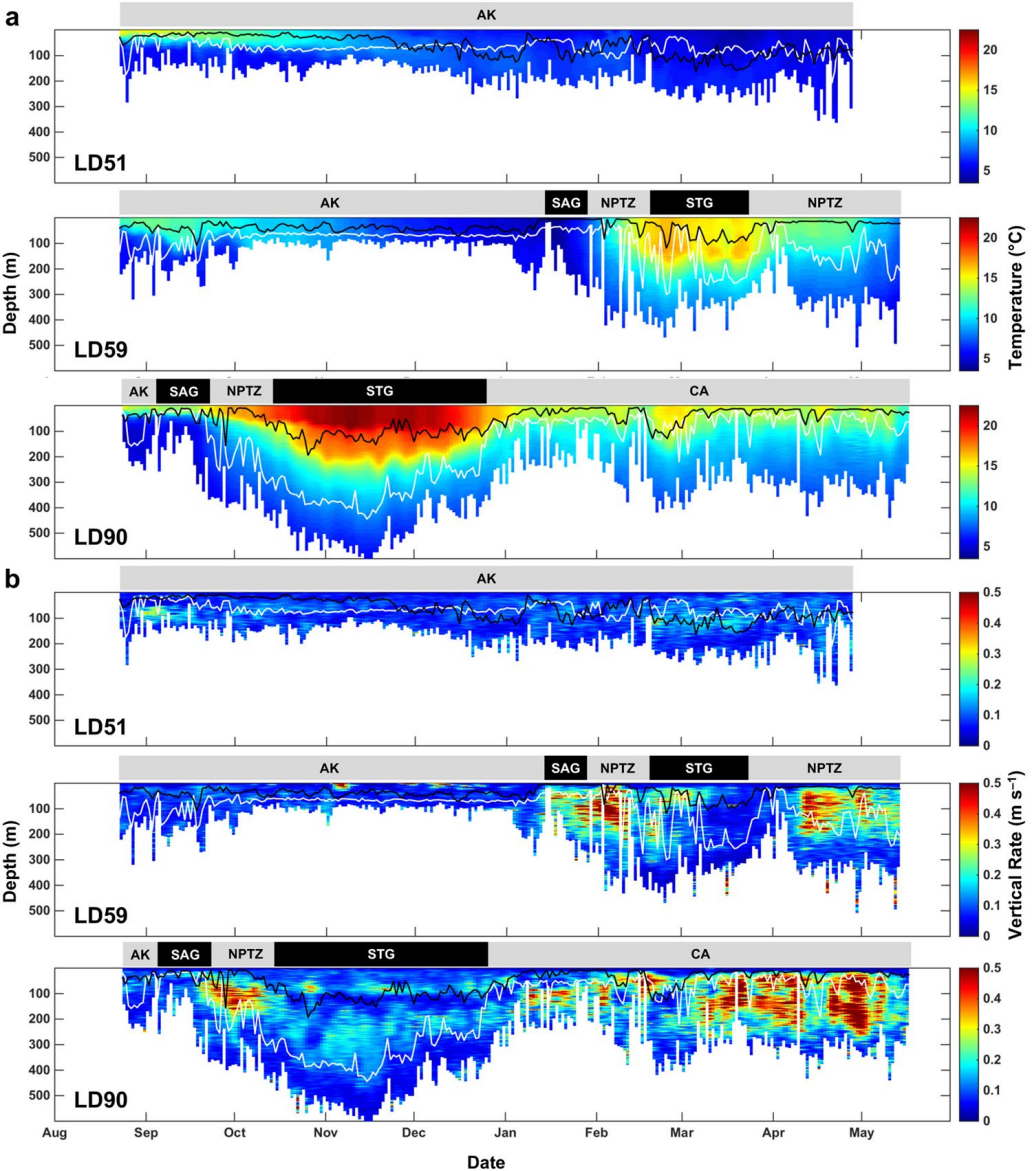
Archival time series of water column thermal structure and vertical displacement rate at depth. (**a**) Water column thermal structure experienced by sharks overwintering in the Alaska Coastal Downwelling (LD51), migrating to the Subtropical Gyre (LD59), and migrating to the California Current (LD90). Water column thermal structure is color-coded by temperature, and bottom limit denotes daily maximum depth. Note that sharks rarely dove below 600 m, but maximum depth was truncated for clearer illustration. (**b**) Vertical rate of displacement at depth experienced by sharks; color-coded by vertical rate. Solid lines denote mean depth during night (black) and day (white). Ticks on x-axis denote the start of the month.

**Figure 5:**
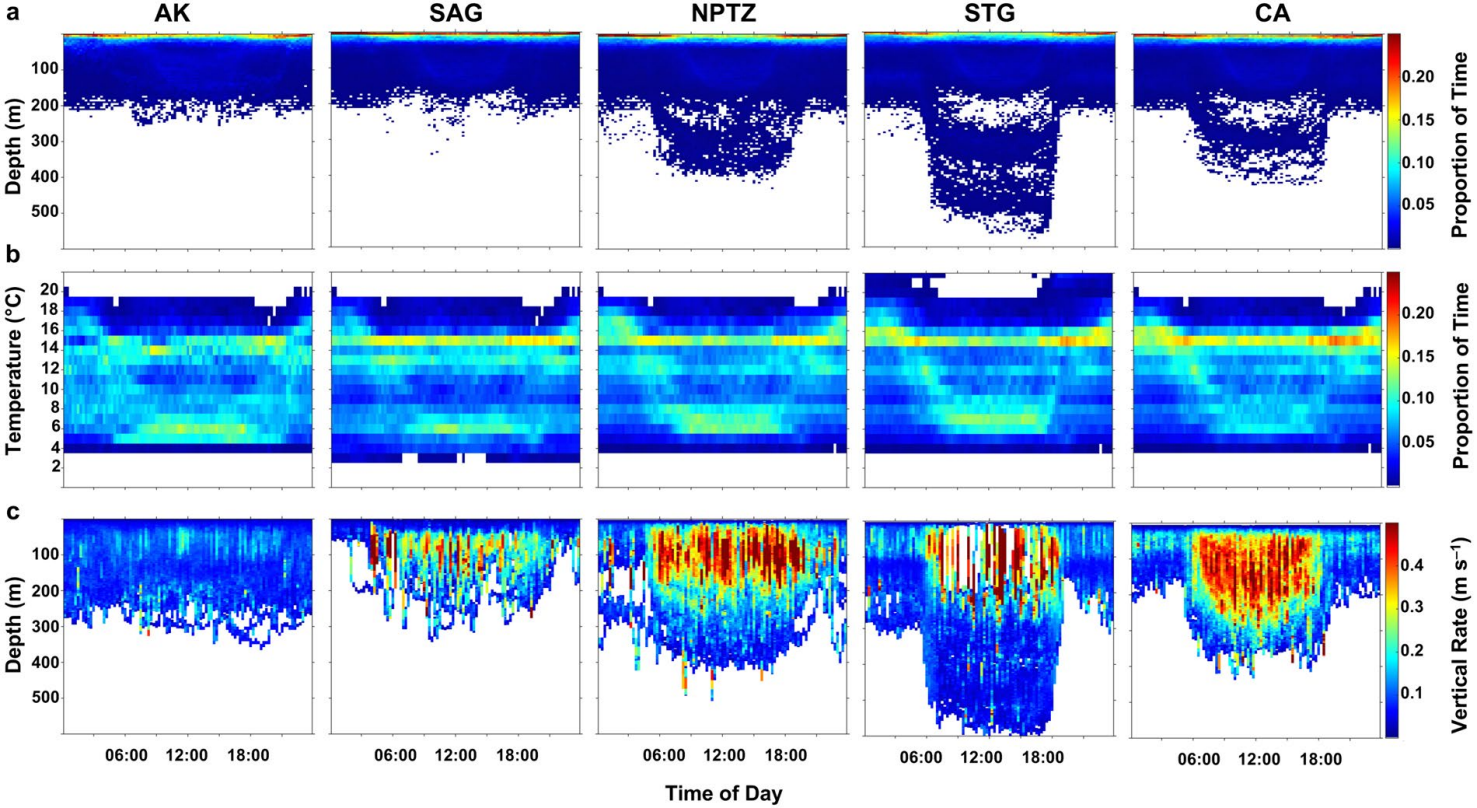
Diel patterns in depth, temperature, and vertical displacement rates. (**a**) Mean proportion of time spent at depth (TAD) and (**b**) temperature (TAT) by time of day for all recovered archival records aggregated by ecoregion. Plots are color-coded by proportion of time. Proportions greater than 0.25 were truncated for clearer illustration and higher proportions were observed. (**c**) Mean vertical displacement rate by depth and time of day for all recovered archival records. Plots are color-coded by vertical displacement rate. Values greater than 0.5 m s^-1^ were truncated for clearer illustration and higher values were observed. Note all eleven sharks (865 total days) with recovered archival records were present in the Alaska Coastal Downwelling (AK); five sharks (212 total days) migrated to the Subarctic Gyre (SAG); two sharks migrated to the North Pacific Transition Zone (NPTZ; 94 total days) and Subtropical Gyre (STG; 107 total days); and one shark (145 total days) migrated to the California Current (CA).

Archival records revealed the depth distribution of salmon sharks changed in a consistent manner in relation to water column thermal structure and exhibited clear diel differences (Figs 4 and 5). Diel patterns were also discernible in thermal habitat use, as sharks primarily occupied warmer waters during shallower night-time distributions and encountered colder temperatures when diving to greater depths during the day (Fig. 5). Shifts in thermal habitat use also reflect seasonal variations in water column thermal structure depending on the timing of each migration.

Salmon sharks in AK generally remained above the thermocline in warm, shallow waters throughout the day when the water column was highly stratified during the summer. As stratification began to break down during the autumn, the daytime distribution of sharks shifted deeper (see Carlisle *et al*.^10^). Vertical displacement rates were largely suppressed while in the cold waters of AK relative to other ecoregions (Figs 4 and 5).

Sharks migrating to the SAG generally remained near the surface (<20 m) where temperatures ranged from 13–15 °C during the autumn to 6–7 °C in the winter (Fig. 5; Supplementary Fig. S2). The shallow distribution of salmon sharks in the SAG was punctuated with rapid vertical movements into deeper water (100–200 m) during the day where sharks encountered temperatures of 4–6 °C (Fig. 5; Supplementary Fig. S2).

Recovered archival records from two sharks (LD59, LD90) that migrated to the NPTZ showed a more apparent diel pattern where they generally used the warmer, shallower part of the water column (0–10 m, 13–17 °C) at night and dove to colder depths during the day (150–350 m, 5–8 °C) (Figs 4 and 5; Supplementary Fig. S3). Consistent high rates of vertical activity were observed during the day while sharks typically exhibited ‘U-shaped’ dive profiles moving between shallow waters and deeper focal depths (Figs 4 and 5; Supplementary Fig. S3).

**Figure 6:**
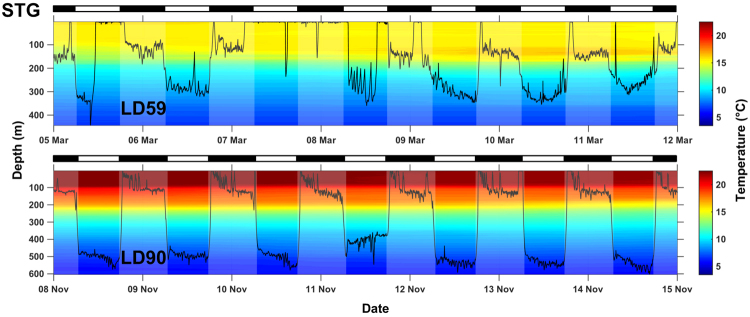
One-week representative depth time series (line) from sharks LD59 and LD90 within the Subtropical Gyre (STG). Background water column thermal structure is color-coded by temperature and night-time is shaded grey. Black and white bars above the time series indicate night and day, respectively.

LD59 and LD90 exhibited a pronounced diel pattern in the STG with distinct changes in depth and temperature distribution (Figs 4, 5 and 6); however, there were marked differences in the vertical distribution of each shark as a result of timing and distance of migration as well as residency time within the STG. LD90 migrated south to 24°N in the STG during autumn for 72 days when SSTs ranged 17–23 °C while LD59 migrated to 31°N in winter when SSTs ranged 14–17 °C for a period of 35 days. The daytime depth distribution of LD90 in the STG was deeper than in the NPTZ; however, vertical activity was less dynamic (Fig. 4). At dawn, LD90 would leave warmer, shallower distributions (100–150 m, 18–19 °C) and descend to colder focal depths (300–550 m, 7–8 °C) where it would remain during daytime hours (Fig. 6). At dusk, LD90 would return to shallower focal depths with only brief forays into surface waters (0–10 m) when temperatures exceeded 18 °C. LD59 exhibited a similar bimodal distribution with shallower daytime focal depths (200–300 m) compared to LD90 (Fig. 6). In contrast, LD59 primarily inhabited the top 5 m during daytime hours for several days while in the STG with infrequent dives to 250–400 m.

A single shark (LD90) with a recovered archival record (270 days) migrated to CA in winter for a period of 145 days. LD90 generally remained in the warm mixed layer during both day and night. Though, a diel pattern was apparent in the vertical behaviour of this shark as it frequently exhibited ‘bounce’ dives or ‘V-shaped’ dive profiles from surface waters (0–10 m, 14–15 °C) to depth (150–300 m, 6–8 °C) during the day (Figs 4 and 5; Supplementary Fig. S4).

### Maximum depth

**Figure 7:**
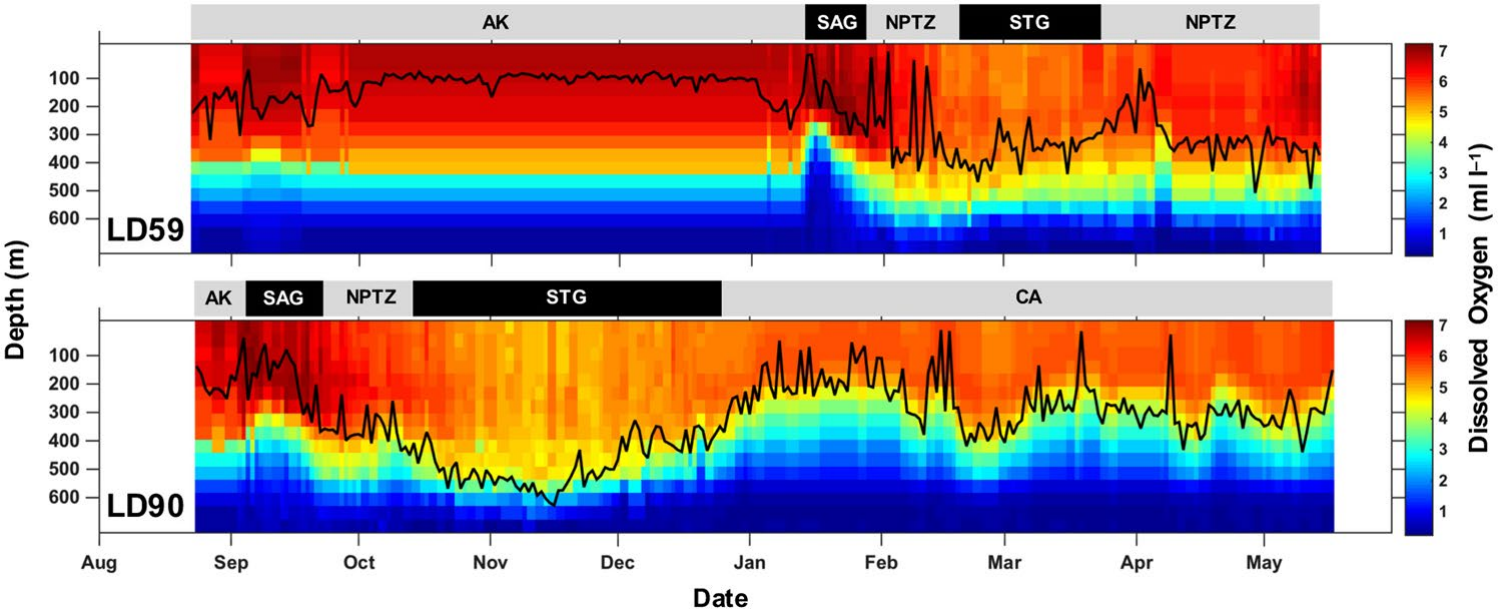
Daily maximum depths (black lines) ascertained from recovered archival records displayed over World Ocean Atlas 2013 monthly mean dissolved oxygen concentrations extracted for each track position for sharks migrating to the Subtropical Gyre (LD59) and California Current (LD90). Ticks on x-axis denote the start of the month.

Salmon sharks progressively made deeper dives as they migrated from Alaskan waters to the STG. Similarly, salmon sharks dove deeper when moving from neritic to offshore waters with the shallowest median maximum depths occurring in AK (136 m) and deepest in the STG (492 m; Supplementary Fig. S5). LD69 had the overall deepest dive of 968 m in the SAG. Median temperatures at the maximum depth of the dives were similar across ecoregions ranging from 6.0 to 7.2 °C with the coldest temperature (1.7 °C) experienced in AK (Supplementary Fig. S5). Median DO concentrations approximated at maximum depth were highest in AK (5.0 ml l^−1^) and lowest in the STG (2.6 ml l^−1^) and CA (2.8 ml l^−1^; Supplementary Fig. S5). Minimum DO concentrations ranged from 0.4 to 0.9 ml l^−1^. Maximum depth was overlaid onto contours of DO concentration along the track for two archival records (LD59, LD90) that made distant migrations offshore (Fig. 7). While migrating outside AK, their maximum depth generally tracked DO isopleths of 4–5 ml l^−1^.

### Model results

Environmental factors and shark size were examined for their effect on the daily proportion of time spent ≥100 m and maximum depth of salmon sharks. Absolute Pearson correlation coefficients were ≤0.56 and variance inflation factors (VIF) were ≤3 suggesting low collinearity between predictor variables. A single model best fit the data for daily proportion of time spent ≥100 m and included all candidate predictor variables except precaudal length (PCL) although residual analyses suggested a departure from normality; therefore, the results must be taken with caution (Supplementary Figs S6 and S7). The full model explained 31.5% of deviance in proportion of time spent ≥100 m by salmon sharks indicating there are additional factors beyond our model explaining a significant portion of their vertical distribution (Table 1). Salmon sharks spent less time below 100 m when migrating offshore of the continental shelf (~200 m) into waters shallower than 3,000 m and when the ILD was deeper than 40 m (Supplementary Fig. S6). Sharks also decreased their proportion of time spent ≥100 m as DO at 100 m declined below 4.3 ml l^−1^. In contrast, salmon sharks spent greater time below 100 m when SST was colder than 11 °C or warmer than 23 °C, log-transformed chl *a* ranged from 0.2 to 2.6 mg m^−3^, and temperatures at 100 m increased above 10 °C.

**Table 1:** Summary statistics from the final GAMM on daily proportion of time spent ≥100 m by salmon sharks.

Term	edf	chi-square	p-value
f(Longitude, Latitude)	28.44	2578.45	<0.00001
f(Bathymetry, m)	3.91	79.76	<0.00001
f(ILD, m)	3.91	217.32	<0.00001
f(SST, °C)	3.88	367.29	<0.00001
f(Log[chl *a*, mg m^−3^])	3.87	51.16	<0.00001
f(Temperature at 100 m, °C)	3.99	1131.90	<0.00001
f(Dissolved Oxygen at 100 m, ml l^−1^)	3.87	143.84	<0.00001

n = 1227, df = 17, adjusted r^2^ = 0.297, AIC_*c*_ = 20327.99, *w* = 0.749, deviance explained = 31.5%.

edf, estimated degrees of freedom.

**Figure 8:**
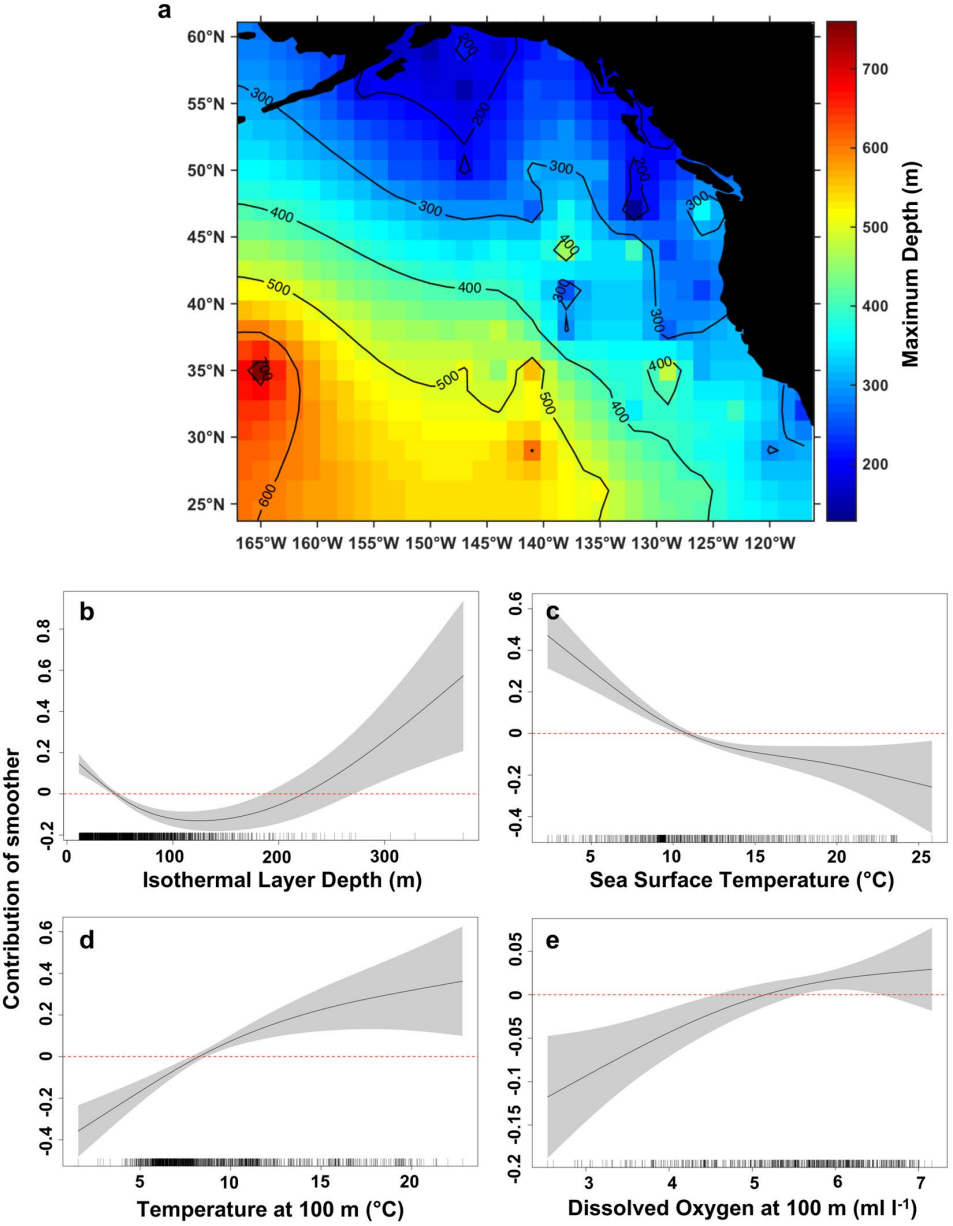
Prediction map and response curves from the final GAMM on daily maximum depth by salmon sharks. (**a**) Model predictions of daily maximum depth from the final GAMM are averaged in a 1.5° × 1.5° grid and plotted in false colour with numbered contours representative of predicted maximum depth. Cells without data are interpolated using a spring metaphor. Map was created using MATLAB R2015a (The MathWorks Inc., Natick, MA, USA, https://www.mathworks.com/products/matlab.html). (**b**–**e**) Estimated response curves (black solid line) of component smooth functions on daily maximum depth from the final GAMM. Shaded areas represent 95% confidence limits of uncertainty in the centred smooth. Vertical axes are partial responses (estimated, centred smooth functions) on the scale of the linear predictor. Ticks on x-axis denote values for which there are data. Positive values on y-axis (above red dashed line) indicate increased maximum depth.

A single model best fit the data for daily maximum depth of salmon sharks and included ILD, SST, temperature at 100 m and DO at 100 m as predictor variables (Fig. 8). Residual analyses suggested no major departures from normality (Supplementary Fig. S8) and the model explained 68.0% of deviance in maximum depth of salmon sharks (Table 2). The maximum depth of salmon sharks increased when the ILD was ≤40 m and ≥220 m, temperatures at 100 m increased and DO at 100 m increased (Fig. 8). Moreover, the maximum depth of salmon sharks shoaled as SSTs increased.

**Table 2:** Summary statistics from the final GAMM on daily maximum depth of salmon sharks.

Term	edf	F-ratio	p-value
f(Longitude, Latitude)	20.14	21.04	<0.00001
f(ILD, m)	3.14	19.53	<0.00001
f(SST, °C)	2.66	18.01	<0.00001
f(Temperature at 100 m, °C)	2.10	17.79	<0.00001
f(Dissolved Oxygen at 100 m, ml l^−1^)	1.71	9.32	0.00457

n = 1740, df = 14, adjusted r^2^ = 0.668, AIC_*c*_ = 18934.33, *w* = 0.633, deviance explained = 68.0%.

edf, estimated degrees of freedom.

## Discussion

This study provides the most comprehensive characterization of vertical habitat use for female salmon sharks across their migratory range in the NEP and revealed distinct differences across disparate oceanographic regions. Combining vertical distribution with high-resolution horizontal movements furthers our understanding of the oceanographic and ecological drivers that influence the spatiotemporal patterns of movement for these top predators. Salmon sharks utilized a broad thermal niche and may experience thermal limits outside their preferred temperature distribution eliciting submergence behaviour, consistent with previous studies^7, 10^; however, this was the first study to demonstrate their ability to tolerate reduced DO concentrations for extended periods of time, especially in the southern extent of their range. Though, the combined effects of cold temperatures and low DO concentrations at shallow depths may restrict the vertical habitat use of salmon sharks or their prey. Moreover, salmon sharks consistently exhibited diel shifts in their patterns of vertical movement and increased utilization of deeper waters in offshore habitats likely reflecting shifts in their foraging ecology across their range.

The depth distributions of salmon sharks changed in association with shifts in water column thermal and DO structure as they moved across the NEP, suggesting that their movements and behaviours were influenced by environmental conditions. Our data confirm that salmon sharks clearly inhabit a broad range of ocean temperatures, ranging from near 0 to 26 °C at the surface; however, the large electronic tagging data set indicates they primarily utilize waters between 6 to 18 °C across their geographic range and are able to tolerate temperature fluctuations of over 20 °C during daily vertical excursions. Salmon sharks overwintering in AK experienced the coldest temperatures, where they inhabit waters <6 °C, and as cold as 2 °C, for months at a time as described in Carlisle *et al*.^10^. This ability to reside in high latitude waters throughout the cold winter months is enabled by the pronounced endothermic physiology inclusive of cardiac specializations of this species^7, 11–13^. The high energetic costs of maintaining a warm body at such cold temperatures have likely been balanced by substantial foraging success on a variety of species such as Pacific herring *Clupea pallasii* and walleye pollock *Gadus chalcogramma* ^9, 10, 18^.

Warm temperatures can have negative impacts on the thermal physiology of large, endothermic fishes as well^19, 20^. Salmon sharks experienced the warmest temperatures (>23 °C) when they were near the surface at night in the STG, but in general they behaviourally position in the water column to remain at slightly deeper, cooler depths (<20 °C) possibly for behavioural thermoregulation. This decrease in surface-oriented behaviour is supported by the marked reduction in Argos positions from Smart Position or Temperature Transmitting Tags (SPOT) when sharks were in the warm waters of the STG^7^. For example, the last Argos position for LD12 was at 31°N with a SST of 17–18 °C before migrating south to the Hawaiian archipelago where the PAT tag popped off on schedule 64 days later during the winter at 22°N when SSTs were 24–25 °C. TAD and TAT data revealed LD12 remained deeper than 100 m primarily inhabiting waters <22 °C following its last Argos position until the PAT tag released. The next SPOT-derived Argos position was 36 days after the release of the PAT tag at 32°N during the spring (see Weng *et al*.^7^) indicating submergence behaviour while migrating through warm SSTs.

The use of deeper waters as a potential thermal refuge for body temperature cooling is comparable to overwintering salmon sharks in AK that use deeper waters that are warmer than surface waters, due to the presence of cold surface inversion layers, as a thermal refuge^10^. For comparison, similar behaviours have been displayed by Atlantic bluefin tuna *Thunnus thynnus* in the Gulf of Mexico where they spend significant time at depth in the thermocline, making brief forays into the warm surface waters (25–28 °C)^20^. Such behaviour is suggestive of an upper thermal limit for salmon sharks at ambient temperatures >20–22 °C. Additional information on salmon shark body core temperatures across their full vertical and geographic range would help to identify potential thermal limits and whether these sharks are actively trying to reduce body temperature and the oxygen demands for the cardiac system. To date, only one implanted archival tag (TDR-MK9, Wildlife Computers, Redmond, WA, USA) has been successfully recovered from a salmon shark that was at liberty for ~3 years before being recaptured by a commercial set net in Alaskan waters. Archival time-series data was recovered for ~1.5 years and the shark undertook two migrations to the STG according to ambient temperature data (Supplementary Fig. S9); however, this tag was not included in the analysis due to light sensor failure. In addition, the tag was implanted in an extraperitoneal position in the abdominal wall and not in the body cavity; therefore, the internal temperature data is not representative of body core temperature. It is noteworthy that this individual reached a maximum depth of >1864 m^10^ exceeding the pressure sensor range for the tag (Supplementary Fig. S9). The near two-fold increase in maximum depth of this individual compared to PAT records (968 m) is likely due to the longer time at liberty, especially in pelagic habitats, and higher resolution of archival time-series data compared to PAT data transmitted to the Argos satellite system.

In addition to occupying a broad thermal range, salmon sharks were able to tolerate low DO concentrations (<1–3 ml l^−1^) for extended periods of time (e.g., up to 9 h in the STG). The ability of salmon sharks to maintain high aerobic muscle performance and maintain a warm body requires elevated metabolic rates and cardiovascular demands^21^ that may preclude high aerobic activity under hypoxic conditions^22^. However, lamnid sharks are known to have high concentrations of red muscle myoglobin^23^ and possess adaptations that enhance oxygen transfer across the gills, increase oxygen transport capacity within the blood, and elevate the delivery rate of oxygenated blood to tissues, suggesting they are capable of entering low DO environments^21, 24–26^. For example, lamnid sharks have a larger gill surface area and thinner gill membrane thickness compared to most other fishes, which enhances oxygen uptake and diffusion capacity at the gills^21, 27^.

There is currently limited to no information on the physiological adaptations of lamnid sharks to hypoxia. However, there are some endothermic fishes that have managed to minimize the trade-offs between high metabolic rates and tolerance for hypoxic conditions. For example, in bigeye tuna *Thunnus obesus* the onset of cardio-respiratory adjustments during acute hypoxia occurs at lower DO concentrations compared to skipjack *Katsuwonus pelamis* or yellowfin tunas *Thunnus albacares* ^28, 29^. In this case, greater hypoxia tolerance is due to enhanced oxygen extraction and transport within the blood^29^ and this unique adaptation allows bigeye tuna to dive to depths where ambient DO is <1.5 ml l^−1^ ^30^, whereas DO concentrations <3.5 ml l^−1^ limits the depth distribution of skipjack and yellowfin tunas^31, 32^. Additional research is needed to measure the hypoxia tolerance of lamnid sharks and identify the physiological specializations that enable them to penetrate into the low oxygen environments of the mesopelagic.

Overall salmon sharks in this study spent the majority of time at relatively high DO concentrations (>5 ml l^−1^), yet were still capable of making excursions to depths with DO concentrations estimated as low as ~0.4 ml l^−1^. This suggests that salmon sharks are able to enter low DO environments (<1–3 ml l^−1^); however, their preferred distribution, or that of their prey, primarily occurs at concentrations >5 ml l^−1^. Salmon sharks were shown to reduce their use of waters below 100 m in the SAG and CA, which are characterized by highly stratified temperature and DO profiles around 100 m relative to other ecoregions. Similarly, salmon sharks primarily utilized habitats shallower than 100 m in AK, where the stratification in temperature and DO is greater than the offshore habitats of the NPTZ and STG, and generally only used waters deeper than 100 m during the winter and spring when the water column became largely well-mixed and cold.

In contrast, salmon sharks spent a considerable amount of time in waters with DO concentrations of 1–3 ml l^−1^ at daytime depths in the STG. The waters of the STG are characterized by weak stratification in temperature and DO, and temperatures experienced at daytime depths (250–550 m) typically ranged from 6–12 °C. Salmon sharks regularly experienced temperatures within this range, typically at shallower depths, across their entire distribution. This suggests that DO concentrations between 1–3 ml l^−1^ may not limit the vertical distribution of salmon sharks when temperatures are >6 °C. In contrast, the vertical distribution of salmon sharks was compressed in AK, SAG and CA, which are characterized by colder temperatures, lower DO concentrations, and sharper gradients below 100 m compared to the offshore habitats of the NPTZ and STG. While salmon sharks overwintering in AK experienced the coldest temperatures (<6 °C) for months at a time, they spent little time in waters with low DO (<3 ml l^−1^). Although salmon sharks can experience waters with cold temperatures (<6 °C) and low DO concentrations (<1–3 ml l^−1^), their depth distribution was generally more restricted when both environmental conditions were limiting at shallow depths, suggestive of vertical habitat compression for salmon sharks or their prey. While characterizing vertical habitat compression in neritic regions such as AK and CA is difficult to analyse due to bathymetric restrictions on vertical movements, the equally compressed vertical distribution of salmon sharks in the pelagic region of the SAG highlights the combined influence of cold temperatures and reduced DO on the vertical habitat use of salmon sharks. Furthermore, models revealed that temperature and DO at depth were both important drivers of vertical distribution as salmon sharks reduced their use of deep waters when temperature and DO at 100 m decreased. The reduced effect of DO in these models is likely due to the use of climatological data in this study instead of *in situ* DO data. With the advent of DO PAT technology^33^, the influence of DO on the vertical distribution of salmon sharks and other wide-ranging species may be better assessed.

The influence of oceanographic conditions on the vertical distribution of salmon sharks is also mediated through its impact on the abundance and distribution of important ectothermic prey species. Due to the unique anatomy and physiology of salmon sharks and sister taxa of the Lamnidae the influence of water temperature and DO on their vertical distributions is likely reduced compared to other species^7, 8, 10^. Salmon sharks have a physiological advantage allowing them to exploit prey resources at higher latitudes more effectively than ectothermic predators^7, 18^. An enhanced endothermic and cardiac capacity also enables salmon sharks to maintain higher levels of activity for longer durations when diving below the mixed layer to exploit deep forage resources more effectively as demonstrated by endothermic tunas^34, 35^. For example, salmon sharks exhibited clear diel patterns in depth distribution diving to deep waters during the day and occupying shallower waters at night within the pelagic habitats of the NPTZ and STG. This bimodal depth distribution suggests an increased reliance on the deep scattering layer (DSL) community^36, 37^ relative to other ecoregions. Several species are known to exhibit similar diel vertical migratory behaviour when moving into offshore habitats including the salmon shark’s congener, the porbeagle shark *Lamna nasus* in the Atlantic Ocean^38^ and waters off New Zealand^39^, and white sharks *Carcharodon carcharias* within the STG^26, 40^. A better understanding of the spatiotemporal patterns in DSL distribution and composition is an important component towards understanding the offshore behaviour and distribution of salmon sharks and other pelagic predators^36, 37, 41^. Furthermore, our results may provide evidence of an important link between salmon sharks and their prey in the DSL community, thus revealing their ecological role in pelagic ecosystems^14^.

Diel shifts in vertical displacement rates were also observed as salmon sharks increased vertical activity during the day. Although salmon sharks exhibited reduced vertical activity in AK relative to other ecoregions, their physiology confers high performance swimming in cold, subarctic waters^13^; therefore, this reduction in vertical activity is more likely a function of cold temperatures impacting the vertical movements and distribution of important ectothermic prey (e.g. salmon). In contrast, high rates of vertical movement were consistently observed during the day in the NPTZ and CA as salmon sharks would make repeated dives below the mixed layer followed by a surface period, though there was a noticeable difference in dive duration and surface intervals between dives. Salmon sharks typically exhibited ‘U-shaped’ dive profiles in the NPTZ whereas ‘bounce’ dives or ‘V-shaped’ dive profiles were more prevalent in CA^42, 43^. U-shaped dives may be representative of marine animals tracking and exploiting aggregated prey (e.g., the DSL) for extended periods, while V-shaped dives are associated with transiting or searching behaviours that may reduce the cost of locomotion^42–44^. Shifts in diving behaviour may reflect changes in the availability of prey resources^45^ and differences in water column stratification among ecoregions, which can influence prey distributions^37, 46^ and the physiology of salmon sharks (e.g., thermoregulation^47^, incurred oxygen debt^48^). Direct measurement of feeding events and body temperatures of tagged sharks across their full range are needed to resolve whether shifts in the vertical distribution and behaviour of salmon sharks serve a thermoregulatory function^47^ or reflect targeting different prey resources and tracking vertically migrating prey^34, 49^.

Characterizing shifts in the vertical distribution of salmon sharks in response to region-specific biological and environmental conditions helps to elucidate the ecosystem role, environmental preferences and physiological capabilities of these top predators. Salmon sharks displayed remarkable plasticity in habitat use and their vertical distribution consistently changed over diel scales and when migrating between disparate ecoregions across multiple years of tagging. The unique anatomy and physiology of salmon sharks enables them to utilize a broad vertical niche and exploit a wide range of thermal and DO environments. This expanded niche provides greater access to seasonally available resources and a potential foraging advantage over ectothermic species^7^. However, the vertical habitat of salmon sharks curtailed when temperature and DO were both limiting at shallow depths. Additional information on thermal and hypoxia tolerance as well as the spatiotemporal patterns of foraging success for this species would help distinguish between changes in vertical distribution and behaviour associated with shifting prey resources or environmental stressors. Understanding patterns of habitat use and environmental preferences of salmon sharks across their full geographic range will foster improved management practices and facilitate the prediction of potential shifts in distribution associated with a changing climate^50^.

## Methods

### Satellite tagging

During July and August of 2002 to 2007, 96 female salmon sharks (Supplementary Table S1) were captured and tagged in Port Gravina, Prince William Sound, Alaska (60.75°N, 146.16°W) as part of the Tagging of Pacific Predators (TOPP) program^1^ using techniques described in Weng *et al*.^7^. All procedures were in accordance with Stanford University Institutional Animal Care and Use Committee protocols and all experimental protocols were approved by the Stanford University Administrative Panel on Laboratory Animal Care (APLAC) under permit APLAC-10765. These sharks had a mean (±s.d) PCL of 210.8 ± 10.0 cm and all were assumed to be mature based on size at maturity estimates^51^.

All sharks were tagged with a PAT tag (PAT1, 2, 3, 4, Mk10, Wildlife Computers, Redmond, WA, USA) and 89 sharks were “double tagged” with a SPOT tag (SPOT1, 2, 3, 4, 5, Wildlife Computers, Redmond, WA, USA). SPOT tags use the Argos satellite system to provide geographic positions with a high degree of resolution when the tag is at the surface. PAT tags archive environmental data (pressure, temperature, light) and are programmed to detach from the tagged animal and float to the surface. Once at the surface, PAT tags transmit summaries of the archived environmental data at user-defined intervals to the Argos satellite system. These summaries are used to reconstruct the movements, habitat use, and environment that the tagged animal experienced, even if the tag is not recovered; however, physical retrieval of the PAT tag provides the entire archived time series permitting more detailed analyses.

### Horizontal movements

Movements were examined using Argos positions from SPOT tags and were linked with associated PAT environmental data for all double-tagged sharks. For PAT tags that successfully transmitted data, the SPOT tags of 8 double-tagged sharks yielded poor Argos positions and 4 sharks were tagged only with PAT tags, so for these 12 sharks locations were obtained using light-based geolocation methods refined by comparing tag-derived and remotely-sensed SST measurements^52^. Bayesian state-space models were fitted to both the Argos- and geolocation-based tracks to account for observation error, regularize shark location estimates through time, and to interpolate over missing observations as described by Block *et al*.^1^ and Winship *et al*.^53^.

The range of salmon sharks in the NEP was divided into ecoregions based on the biogeographic provinces defined by Longhurst^54^. Neritic regions include the Alaska Coastal Downwelling Province (AK) and the California Current Province (CA). Pelagic regions include the Pacific Subarctic Gyre Province (SAG), North Pacific Polar Front Province, which is analogous to the North Pacific Transition Zone (NPTZ), and the Pacific Tropical Gyre Province, which is analogous to the Subtropical Gyre (STG).

### Data processing

Of the 96 PAT tags deployed, 67 tags transmitted data and 29 never reported or transmitted no data (Supplementary Table S1). Analyses of the data revealed that 5 of the reporting tags were associated with mortality events within 24 h of release. Eleven PAT tags were recovered providing the capacity to analyse archival data (summarized below). The PAT tags transmitted depth (TAD) and temperature (TAT) data summarized into 4-, 6-, 12- or 24-h histograms with user-defined bins, PDT data including minimum and maximum depth for each summary period, daily SST, and profiles of dawn and dusk light levels for geolocation. PAT1 tags (n = 5) deployed in 2002 that transmitted usable data did not log and transmit daily SST; therefore, SST was calculated from transmitted PDT data using the mean temperatures recorded at depths ≤5 m. Bin structure varied across tags, so to allow for comparisons across different programming schemes, TAD and TAT bins were combined according to common bins. The TAT binning scheme of one tag (LD6) precluded its inclusion in analysis of thermal habitat use. TAD, TAT and PDT data were pooled into 24-h intervals and only days with complete 24 h records were aggregated for analysis. Data from the first 24 h were not included in the analysis to reduce potential effects of capture and tagging^55^.

In addition to the 62 transmitted PAT records from salmon sharks, archival records from 11 recovered PAT tags were obtained. Archival records recorded data at higher resolutions (2 to 120 s) depending upon programming, and were analysed in place of transmitted records for 8 tags (Supplementary Table S1). Three archival records were incorporated from recovered PAT tags that did not successfully transmit, yielding a total of 65 individual PAT tag records spanning 2002 to 2008 with 10,431 days of data. For comparative analyses among recovered tags, data were down-sampled to the longest sampling rate (120 s). When analysed together with the transmitted PAT data, archival time-series data were aggregated using the same TAD and TAT bin structure. Note that 8 archival records (LD3, LD20, LD22, LD28, LD31, LD51, LD59, LD91) were presented in detail in Carlisle *et al*.^10^ that focused on behaviours within the neritic waters of Alaska. We include them in this study to allow for a full comparison of vertical distribution across the range of salmon sharks in the NEP. The thermal structure of the water column was visualized along the full track of each archival record as described in Carlisle *et al*.^10^. Vertical rates of movement (i.e., displacement rates) were calculated by dividing the depth difference between successive points by the sampling interval and plotted in a similar fashion as water column thermal structure.

### Diel patterns

It was not possible to examine diel patterns from PAT tags programmed for 12- or 24-h histograms, which prevented using the majority of tags that transmitted usable data; therefore, archived time-series data from recovered tags were used to investigate diel patterns in depth, temperature, and vertical displacement rates. To divide each 24-h period into day or night, sunrise and sunset times were determined for each daily position using the NOAA Solar Calculator (http://www.esrl.noaa.gov/gmd/grad/solcalc/). To describe diel patterns of depth and thermal habitat use, TAD and TAT was calculated at 15 min increments for every 24 h period, then aggregated by ecoregion as described in Carlisle *et al*.^10^. Diel patterns in vertical displacement rates were calculated as described in the previous section.

### Environmental data

Environmental variables were extracted along tagged salmon shark tracks to characterize oceanographic conditions the sharks experienced. Bathymetry was extracted from the ETOPO1 Global Relief Model^56^ (0.1° resolution) and 8-day composite chl *a* concentration was extracted from SeaWifs (0.1° resolution) for 2002 and Aqua MODIS (0.05° resolution) for 2003–2008 using Xtractomatic tools (http://coastwatch.pfel.noaa.gov/xtracto/) implemented in R^57^. We included the Bayesian state-space model error ellipse from each daily position estimate to calculate a mean value using Xtractomatic tools. Daily SST was derived from PAT tags. ILD, a proxy for mixed layer depth, was calculated from PDT data following the method described in Kara *et al*.^58^ with a threshold value of 0.8 °C.

Dissolved oxygen concentrations experienced at depth were approximated by extracting data from the World Ocean Atlas 2013 (WOA13)^59^. The WOA13 provides global, 1° gridded, objectively analysed climatological fields of environmental parameters. Monthly DO concentration profiles (statistical mean) were extracted for each track position. Since the bin structure of the TAD data did not correspond to the bin structure of WOA13 DO concentrations, we reconstructed continuous depth distributions for TAD and WOA13 DO concentrations for each day by interpolating across the binned depth data, assuming that the sharks were uniformly distributed within each depth bin according to methods described in Carlisle *et al*.^60^, using the daily maximum depth recorded by the tag as the maximum depth of the reconstructed depth distribution. We used the interpolated depth distributions to assign each depth a DO concentration and estimate time spent at different oxygen concentrations (TAO).

### Modelling depth distribution

We used generalized additive mixed models (GAMMs) to determine which factors influence the vertical distribution of salmon sharks. Response variables included daily proportion of time spent ≥100 m to investigate the use of deeper habitats and daily maximum depth to investigate the full depth range utilized by salmon sharks. We incorporated location, PCL, bathymetry, temperature at 100 m, DO at 100 m, ILD, SST, and log-transformed chl *a* as candidate predictor variables. Prior to model fitting, data exploration was carried out per Zuur *et al*.^61^. Collinearity between candidate predictor variables was assessed with Pearson correlation coefficients and VIFs using the ‘corvif’ function (http://www.highstat.com/book2.htm ^61, 62^) in R. Since observations were repeated measures collected from the same individuals, we modelled individual sharks as a random effect. To account for spatial autocorrelation, location was included as a fixed covariate using an isotropic thin plate regression spline of latitude and longitude in each model.

Daily proportion of time spent ≥100 m was modelled using a binomial distribution with a logit link function. Proportional values were converted to the ratio of ‘successes’ (proportion of time spent ≥100 m) to ‘failures’ (proportion of time spent <100 m) per day by multiplying proportional values by 24 h and rounding to the nearest integer. Maximum depth was modelled using a gamma distribution with a log link function. Models were explored using unrestricted smooths, but final models limited the basis (*k*) used to represent the smooth terms at 5^63^. GAMMs were constructed in R using the ‘gamm4’ package^64^ and model selection was based upon an information-theoretic approach through minimization of the Akaike Information Criterion corrected for small sample size (AIC_*c*_)^65^ using the ‘MuMIn’ package^66^. Models with substantial support were selected based on a ΔAIC_*c*_ < 2 from the model with the lowest AIC_*c*_ and included in model averaging based on Akaike weights (*w*)^65^. A single best model containing only predictor variables with high relative importance was used for graphical representation of smoothed terms, calculation of deviance explained, and evaluation of model fitness and adherence to statistical assumptions of residuals.

## Electronic supplementary material

Supplementary Information
