# Supplementary material for: Oceanographic drivers of the vertical distribution of a highly migratory, endothermic shark
Source: Sci Rep. 2017 Sep 5;7:10434. doi: 10.1038/s41598-017-11059-6 (PMC5585177; doi:10.1038/s41598-017-11059-6)
Supplement: Supplementary file 1 — Supplementary Information [file 41598_2017_11059_MOESM1_ESM.pdf]

Supplementary Information

Oceanographic drivers of the vertical distribution of a highly migratory, endothermic shark

Daniel M. Coffey[1,2*], Aaron B. Carlisle[3], Elliott L. Hazen[4] and Barbara A. Block[3]

[1]Tuna Research and Conservation Center, Monterey Bay Aquarium, Monterey, CA, USA
[2]Present Address: Hawaii Institute of Marine Biology, University of Hawaii at Manoa, Kaneohe, HI, USA
[3]Hopkins Marine Station, Stanford University, Pacific Grove, CA, USA
[4]Environmental Research Division, Southwest Fisheries Science Center, National Oceanic and Atmospheric Administration, Monterey, CA, USA

[*]Corresponding author: dcoffey@hawaii.edu

Table S1. Female salmon sharks *Lamna ditropis* tagged in Port Gravina, Prince William Sound, Alaska.

Shark ID	PCL (cm)	Tag Type(s)	Tagging Date	Pop-up Date	Pop-up Location	DAL	Summary Interval (h)	TAD	TAT	PDT	Min/Max Depth	SST
LD1	207	PAT1/SPOT1	14 Jul 2002	14 Nov 2002	41.65°N, 124.92°W	123	24	102	93	103	112	109[e]
LD2	196	PAT1/SPOT1	14 Jul 2002	14 Nov 2002	44.88°N, 124.37°W	123	24	62	54	80	110	95[e]
LD3[b,d]	208	PAT1/SPOT1	15 Jul 2002	14 Nov 2002	54.96°N, 160.51°W	122	24	120	120	120	120	120
LD4	216	PAT1/SPOT1	15 Jul 2002	13 Jan 2003	60.78°N, 146.69°W	182	24			146	99	145[e]
LD5	218	PAT1/SPOT1	16 Jul 2002	13 Jan 2003	57.32°N, 133.67°W	181	24	89	80	106	126	119[e]
LD6	214	PAT1	17 Jul 2002	15 Sep 2002	60.49°N, 147.00°W	60	6	53		3	40	58[e]
LD7[a]	218	SPOT2/PAT2	17 Jul 2002				24					
LD8	216	SPOT2/PAT3	15 Aug 2003	16 Feb 2004	35.48°N, 135.79°W	185	12	26	21	42	115	134
LD9	201	SPOT2/PAT3	15 Aug 2003	20 Nov 2003	28.62°N, 149.18°W	97	12	56	40	68	84	76
LD10	220	SPOT2/PAT3	17 Aug 2003	20 May 2004	59.60°N, 144.65°W	277	12			104	184	255
LD11[a]		SPOT2/PAT3	17 Aug 2003				12					
LD12	219	SPOT3/PAT3	18 Aug 2003	20 Feb 2004	22.27°N, 160.24°W	186	12	70	60	100	153	99
LD13	208	SPOT3/PAT3	18 Aug 2003	20 Feb 2004	58.47°N, 147.13°W	186	12	34	22	45	110	134
LD14	216	SPOT3/PAT3	18 Aug 2003	20 Feb 2004	43.04°N, 134.24°W	186	12	63	52	85	136	150
LD15	218	SPOT3/PAT3	19 Aug 2003	20 Feb 2004	31.53°N, 142.76°W	185	12	45	43	83	131	162

Shark ID	PCL (cm)	Tag Type(s)	Tagging Date	Pop-up Date	Pop-up Location	DAL	Summary Interval (h)	TAD	TAT	PDT	Min/Max Depth	SST
LD16[a]	217	SPOT3/PAT3	19 Aug 2003				12					
LD17	219	PAT4/SPOT4	11 Jul 2004	10 Apr 2005	42.45°N, 124.75°W	273	12	18	18	47	97	81
LD18	222	PAT4/SPOT4	11 Jul 2004	11 Apr 2005	33.96°N, 120.81°W	274	12			5	12	13
LD19	212	PAT4/SPOT4	11 Jul 2004	08 Nov 2004	59.94°N, 148.00°W	120	12					1
LD20[d]	218	PAT4/SPOT4	11 Jul 2004	15 Aug 2004	60.69°N, 146.44°W	35	6	22	22	22	22	22
LD21	188	PAT4/SPOT4	12 Jul 2004	08 Jan 2005	57.17°N, 152.83°W	180	12	13	14	33	69	50
LD22[a,d]	214	PAT4/SPOT4	12 Jul 2004	08 Aug 2004	60.34°N, 146.72°W	27	12	26	26	26	26	26
LD23	221	PAT4/SPOT4	12 Jul 2004	12 Apr 2005	40.91°N, 144.83°W	274	12	5	5	15	46	73
LD24	214	PAT4/SPOT4	12 Jul 2004	09 Apr 2005	60.60°N, 147.29°W	271	12	31	20	87	168	146
LD25[a]	224	PAT4	13 Jul 2004				2					
LD26	215	PAT4/SPOT4	13 Jul 2004	09 Jan 2005	56.90°N, 153.10°W	180	6			2	5	32
LD27	210	PAT4	14 Jul 2004	02 Mar 2005	59.11°N, 152.62°W	231	12	13	9	17	62	77
LD28[d]	224	PAT4/SPOT4	13 Jul 2004	09 Jan 2005	59.29°N, 147.11°W	180	6	161	161	161	161	161
LD29	202	PAT4/SPOT4	13 Jul 2004	09 Jan 2005	34.18°N, 123.34°W	180	6			1	16	77
LD30[a]	216	PAT4/SPOT4	13 Jul 2004				6					
LD31[a,d]	207	PAT4/SPOT4	13 Jul 2004	28 Aug 2004	60.72°N, 146.11°W	46	12	41	41	41	41	41

Table S1. continued.

Shark ID	PCL (cm)	Tag Type(s)	Tagging Date	Pop-up Date	Pop-up Location	DAL	Summary Interval (h)	TAD	TAT	PDT	Min/Max Depth	SST
LD32	223	PAT4/SPOT4	13 Jul 2004	09 Jan 2005	57.90°N, 154.13°W	180	12	4	2	11	32	35
LD33[a]	211	PAT4/SPOT4	13 Jul 2004				12					
LD34[a]	229	PAT4/SPOT4	14 Jul 2004				12					
LD35	218	PAT4/SPOT4	14 Jul 2004	12 Mar 2005	30.12°N, 134.62°W	241	12	64	62	83	115	64
LD36[b]	212	PAT4/SPOT4	15 Jul 2004				12					
LD37	210	PAT4	14 Jul 2004	11 Nov 2004	57.99°N, 152.06°W	120	12	11	6	27	61	59
LD38	208	PAT4	15 Jul 2004	08 Jan 2005	45.63°N, 164.40°W	177	12	27	21	56	121	76
LD39	220	PAT4/SPOT5	20 Aug 2005	06 Aug 2006	60.66°N, 146.32°W	351	12	70	57	125	215	275
LD40	191	PAT4/SPOT5	21 Aug 2005	21 May 2006	60.32°N, 146.61°W	273	12	24	19	16	65	186
LD41	208	PAT4/SPOT5	21 Aug 2005	22 Aug 2006	54.07°N, 133.17°W	366	12	33	26	65	171	269
LD42[a]	221	PAT4/SPOT5	21 Aug 2005				12					
LD43	209	PAT4/SPOT5	21 Aug 2005	21 May 2006	60.12°N, 146.65°W	273	12	129	118	87	177	185
LD44	200	PAT4/SPOT5	21 Aug 2005	26 Aug 2005	60.66°N, 146.33°W	5	12					
LD45[d]	217	PAT4/SPOT5	21 Aug 2005	21 Sep 2005	53.29°N, 137.87°W	31	12	31	31	31	31	31
LD46	211	PAT4/SPOT5	21 Aug 2005	23 Apr 2006	37.86°N, 123.38°W	245	12	36	37	25	135	204
LD47[b]	217	PAT4/SPOT5	22 Aug 2005				12					

Table S1. continued.

Shark ID	PCL (cm)	Tag Type(s)	Tagging Date	Pop-up Date	Pop-up Location	DAL	Summary Interval (h)	TAD	TAT	PDT	Min/Max Depth	SST
LD48	198	PAT4/SPOT5	22 Aug 2005	21 May 2006	60.31°N, 146.63°W	272	12	122	107	98	168	210
LD49[a]	198	PAT4/SPOT5	22 Aug 2005				12					
LD50	210	PAT4/SPOT5	22 Aug 2005	02 May 2006	36.53°N, 138.60°W	253	12	56	44	38	120	156
LD51[d]	206	PAT4/SPOT5	22 Aug 2005	21 May 2006	60.72°N, 147.53°W	272	12	249	249	249	249	249
LD52[a]	202	PAT4/SPOT5	22 Aug 2005				12					
LD53	200	PAT4/SPOT5	22 Aug 2005	21 May 2006	34.01°N, 121.11°W	272	12	42	34	21	148	211
LD54	212	PAT4/SPOT5	23 Aug 2005	20 Sep 2005	60.72°N, 146.13°W	28	12					
LD55	211	PAT4/SPOT5	23 Aug 2005	28 Sep 2005	52.88°N, 132.32°W	36	12	25	23	20	23	30
LD56[b]	209	PAT4/SPOT5	23 Aug 2005				12					
LD57	190	MK10/SPOT5	24 Aug 2005	20 Nov 2005	56.92°N, 137.18°W	88	12	38	34	25	41	37
LD58[b]	209	MK10/SPOT5	24 Aug 2005				12					
LD59[d]	212	MK10/SPOT5	20 Aug 2006	16 May 2007	45.80°N, 142.53°W	269	12	265	265	265	265	265
LD60	212	MK10/SPOT5	20 Aug 2006	30 Aug 2006	60.70°N, 146.17°W	10	12					
LD61[b]	211	MK10/SPOT5	20 Aug 2006									
LD62[a]	192	MK10/SPOT5	20 Aug 2006				12					
LD63[b]	233	MK10/SPOT5	21 Aug 2006				12					

Table S1. continued.

Shark ID	PCL (cm)	Tag Type(s)	Tagging Date	Pop-up Date	Pop-up Location	DAL	Summary Interval (h)	TAD	TAT	PDT	Min/Max Depth	SST
LD64[a]	199	MK10/SPOT5	21 Aug 2006				12					
LD65	230	MK10/SPOT5	21 Aug 2006	28 Aug 2006	60.72°N, 146.11°W	7	12					
LD66[b]	227	MK10/SPOT5	21 Aug 2006				24					
LD67[a]	206	MK10/SPOT5	21 Aug 2006				24					
LD68[b]	211	MK10/SPOT5	21 Aug 2006				12					
LD69	208	MK10/SPOT5	21 Aug 2006	19 May 2007	46.32°N, 137.52°W	271	12	103	103	81	138	71
LD70[a]	215	MK10/SPOT5	22 Aug 2006				24					
LD71	210	MK10/SPOT5	22 Aug 2006	25 Sep 2006	56.96°N, 137.72°W	34	24	30	30	27	30	23
LD72	200	MK10/SPOT5	22 Aug 2006	23 Apr 2007	30.72°N, 118.58°W	244	24	7	7	3	3	3[e]
LD73	204	MK10/SPOT5	22 Aug 2006	14 Feb 2007	39.53°N, 135.09°W	176	24	155	156	132	154	53
LD74[b]	218	MK10/SPOT5	22 Aug 2006				24					
LD75[b]	205	MK10/SPOT5	23 Aug 2006				12					
LD76[d]	219	MK10/SPOT5	23 Aug 2006	24 Oct 2006	56.18°N, 151.25°W	62	12	61	61	61	61	61
LD77	197	MK10/SPOT5	23 Aug 2006	20 Apr 2007	30.59°N, 118.14°W	240	12	39	40	18	98	22
LD78	198	MK10/SPOT5	24 Aug 2006	21 May 2007	32.18°N, 120.79°W	270	12	67	67	33	104	32
LD79	201	MK10/SPOT5	24 Aug 2006	03 Sep 2006	60.72°N, 146.11°W	10	12	5	5	2	5	3

Table S1. continued.

Shark ID	PCL (cm)	Tag Type(s)	Tagging Date	Pop-up Date	Pop-up Location	DAL	Summary Interval (h)	TAD	TAT	PDT	Min/Max Depth	SST
LD80	214	MK10/SPOT5	24 Aug 2006	22 Mar 2007	32.93°N, 137.55°W	210	12	95	95	48	91	95
LD81	241	MK10/SPOT5	25 Aug 2006	26 Sep 2006	46.15°N, 132.27°W	32	12	21	21	6	21	17
LD82	216	MK10/SPOT4	25 Aug 2006	21 Feb 2007	43.36°N, 144.27°W	180	12	95	95	64	107	38
LD83	213	MK10/SPOT4	25 Aug 2006	21 Feb 2007	38.08°N, 125.63°W	180	12	118	118	74	128	114
LD84	198	MK10/SPOT4	26 Aug 2006	23 Apr 2007	37.05°N, 122.75°W	240	12	91	90	53	140	110
LD85	195	MK10/SPOT5	21 Aug 2007	17 Dec 2007	53.87°N, 160.95°W	118	24	107	96	94	113	113
LD86[a]	221	MK10	21 Aug 2007				24					
LD87	208	MK10/SPOT5	22 Aug 2007	20 Sep 2007	60.73°N, 146.12°W	29	12	23	23	8	23	24
LD88	205	MK10	22 Aug 2007	28 Aug 2007	60.72°N, 146.13°W	6	12					
LD89	219	MK10/SPOT5	22 Aug 2007	18 Feb 2008	31.83°N, 120.78°W	180	12	61	46	42	90	127
LD90[d]	217	MK10/SPOT5	23 Aug 2007	19 May 2008	35.59°N, 121.20°W	270	24	268	268	268	268	268
LD91[d]	192	MK10/SPOT5	23 Aug 2007	19 Feb 2008	59.70°N, 139.49°W	180	12	179	179	179	179	179
LD92	218	MK10/SPOT5	23 Aug 2007	05 Dec 2007	60.82°N, 147.22°W	104	4	47	50	10	49	101
LD93	200	MK10/SPOT5	24 Aug 2007	05 Jan 2008	59.90°N, 148.06°W	134	24	123	110	116	130	118
LD94	195	MK10/SPOT5	24 Aug 2007	20 Apr 2008	61.07°N, 148.13°W	240	4	50	50	14	63	120
LD95	204	MK10/SPOT5	24 Aug 2007	04 Mar 2008	60.75°N, 146.93°W	193	12	131	131	92	155	170

Table S1. continued.

Shark ID	PCL (cm)	Tag Type(s)	Tagging Date	Pop-up Date	Pop-up Location	DAL	Summary Interval (h)	TAD	TAT	PDT	Min/Max Depth	SST
LD96[a]	214	MK10/SPOT5	24 Aug 2007				24					

PCL, precaudal length; DAL, days at liberty; TAD, time-at-depth histogram; TAT, time-at-temperature histogram; PDT, profile of depth and temperature; SST, sea surface temperature

[a]Never reported

[b]Reported, but transmitted no data

[c]Post-release mortality

[d]Recovered tag

[e]SSTs calculated from transmitted PDT data using the mean temperatures recorded at depths ≤5 m

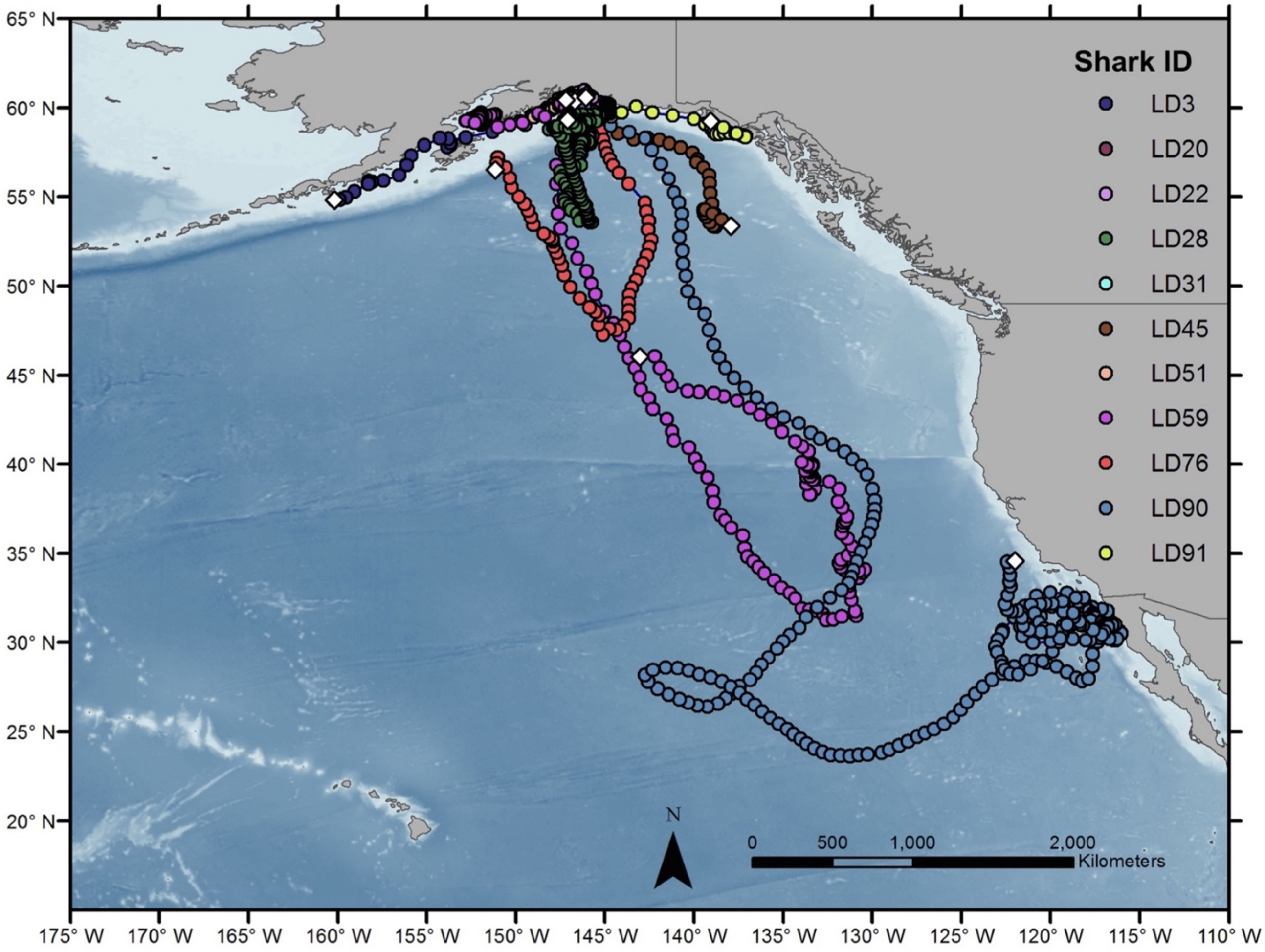

Figure S1. Daily mean Bayesian state-space model position estimates (○) and pop-up/recovery location of PAT tags (◇) corresponding to recovered PAT tags. Only positions that overlap temporally with recovered PAT records are shown. Argos positions are colour-coded by individually tagged sharks. Map was created using ArcGIS 10.2 (ESRI Inc., Redland, CA, USA, http://desktop.arcgis.com/en/).

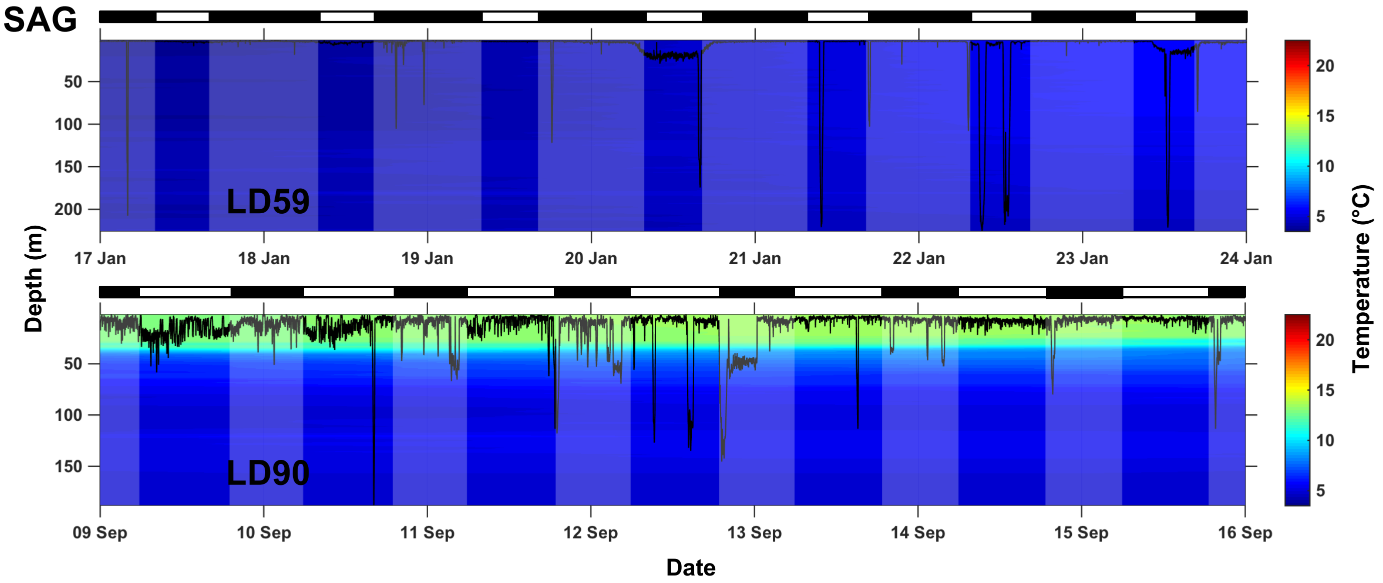

Figure S2. One-week representative depth time series (line) from sharks LD59 and LD90 within the Subarctic Gyre (SAG). Background water column thermal structure is colour-coded by temperature and night-time is shaded grey. Black and white bars above the time series indicate night and day, respectively.

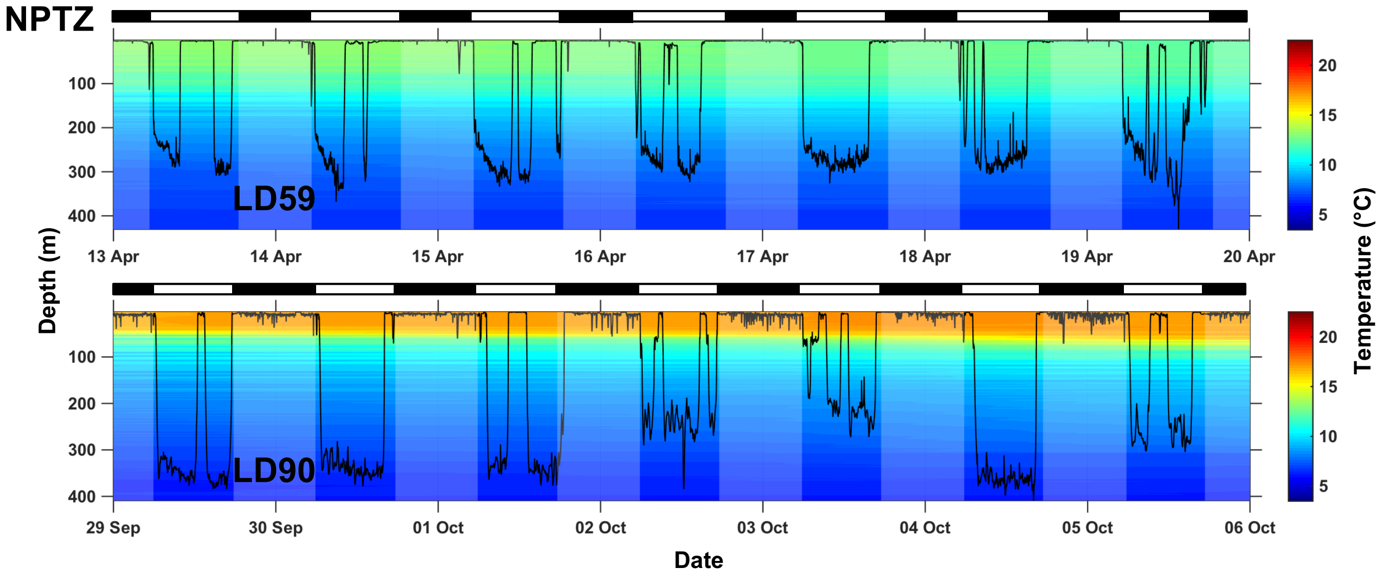

Figure S3. One-week representative depth time series (line) from sharks LD59 and LD90 within the North Pacific Transition Zone (NPTZ). Background water column thermal structure is colour-coded by temperature and night-time is shaded grey. Black and white bars above the time series indicate night and day, respectively.

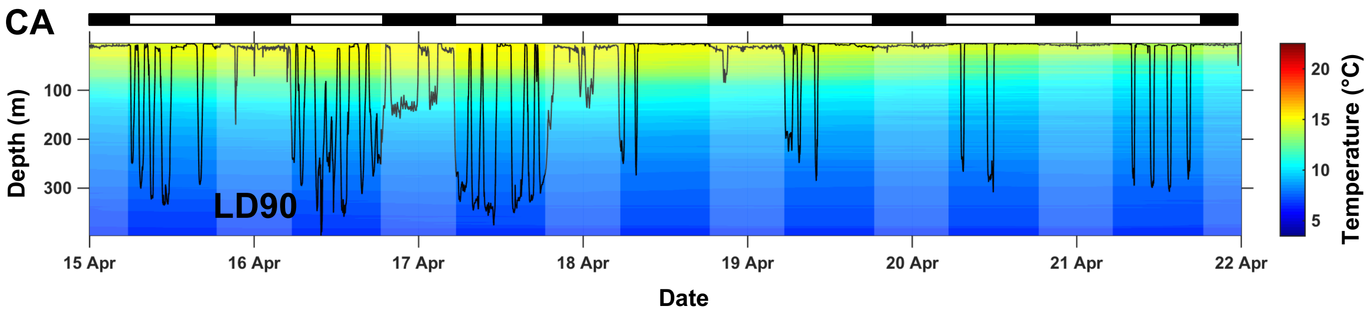

Figure S4. One-week representative depth time series (line) from shark LD90 within the California Current (CA). Background water column thermal structure is colour-coded by temperature and night-time is shaded grey. Black and white bars above the time series indicate night and day, respectively.

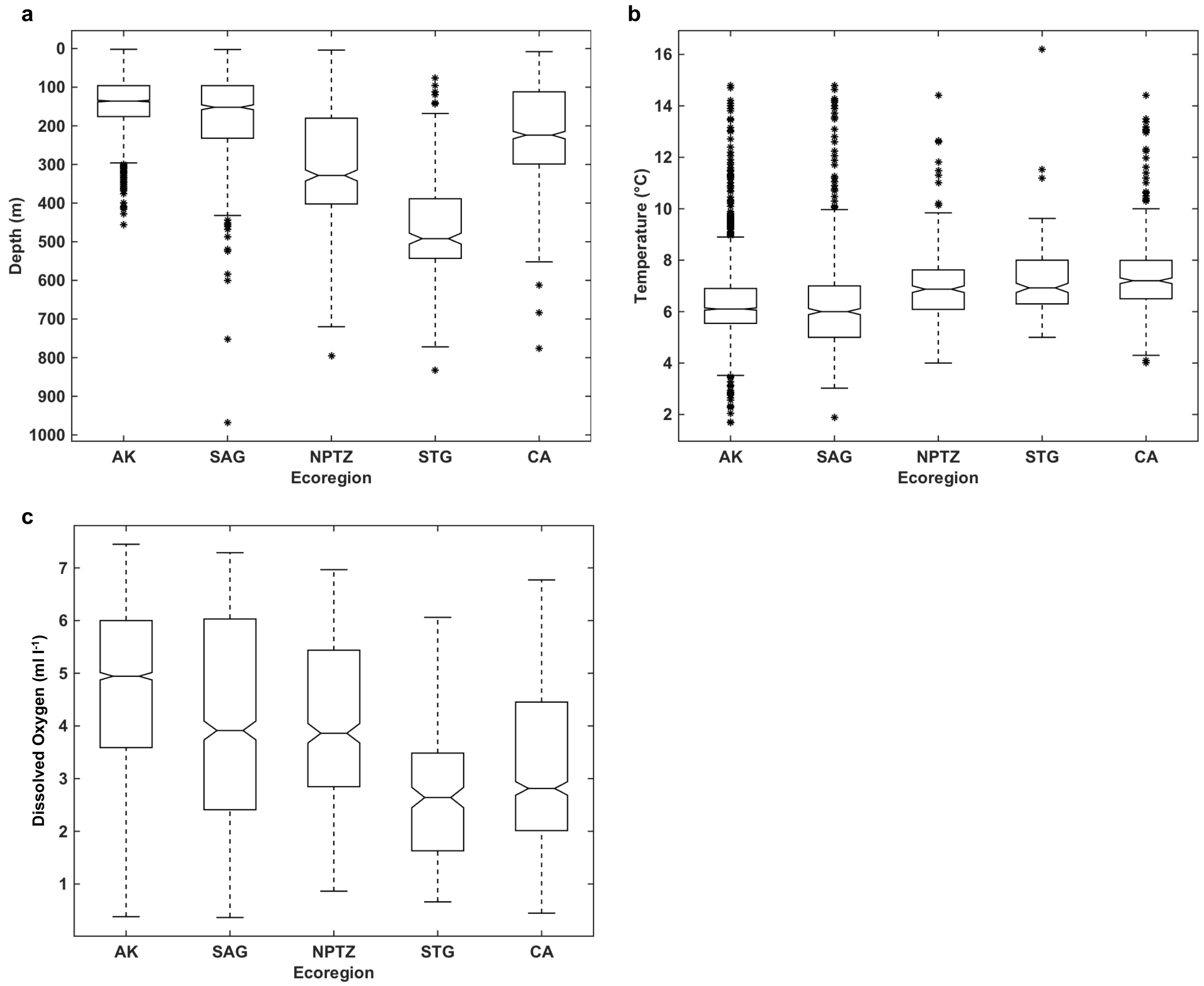

Figure S5. Distribution of daily (**a**) maximum depth (n = 6532), (**b**) temperature at maximum depth (n = 4068), and (**c**) dissolved oxygen concentrations approximated at maximum depth (n = 5441). Box denotes median and interquartile range (IQR, 25th to 75th percentiles); notch denotes the 95% confidence intervals for the medians; whiskers denote points within IQR × 1.5 of the ends of the box; points denote outliers.

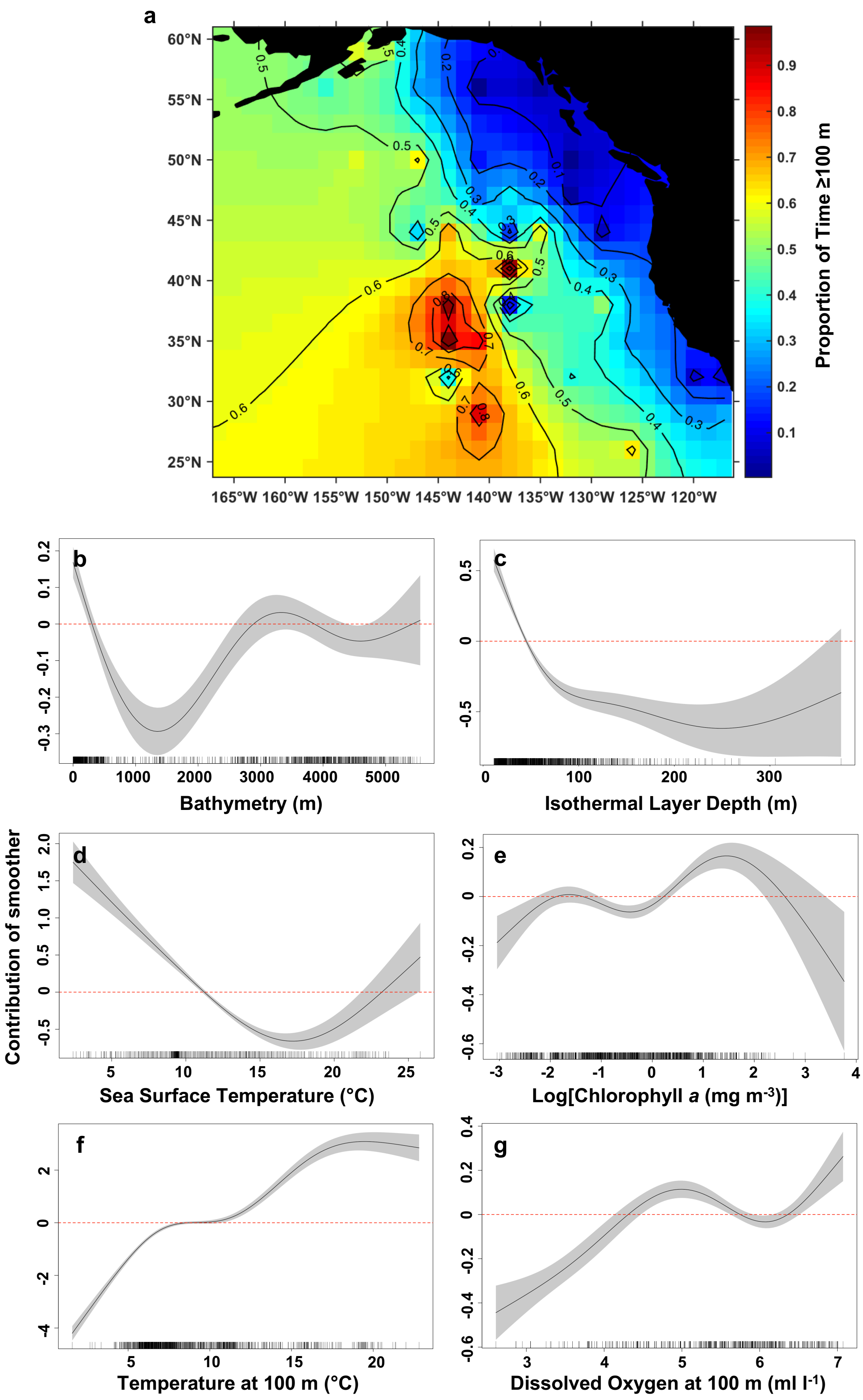
a
60°N
55°N
50°N
45°N
40°N
35°N
30°N
25°N
165°W 160°W 155°W 150°W 145°W 140°W 135°W 130°W 125°W 120°W
Proportion of Time ≥100 m
0.1 0.2 0.3 0.4 0.5 0.6 0.7 0.8 0.9
b
Bathymetry (m)
c
Isothermal Layer Depth (m)
Contribution of smoother
d
Sea Surface Temperature (°C)
e
Log[Chlorophyll a (mg m-3)]
f
Temperature at 100 m (°C)
g
Dissolved Oxygen at 100 m (ml l-1)

Figure S6. (**a**) Model predictions of daily proportion of time spent ≥100 m are averaged in a 1.5° × 1.5° grid and plotted in false colour with numbered contours representative of proportion of time spent ≥100 m. Cells without data are interpolated using a spring metaphor. Map was created using MATLAB R2015a (The MathWorks Inc., Natick, MA, USA, https://www.mathworks.com/products/matlab.html). (**b-g**) Estimated response curves (black solid line) of component smooth functions on daily proportion of time spent ≥100 m. Shaded areas represent 95% confidence limits of uncertainty in the centred smooth. Vertical axes are partial responses (estimated, centred smooth functions) on the scale of the linear predictor. Ticks on x-axis denote values for which there are data. Positive values on y-axis (above red dashed line) indicate increased proportion of time spent ≥100 m.

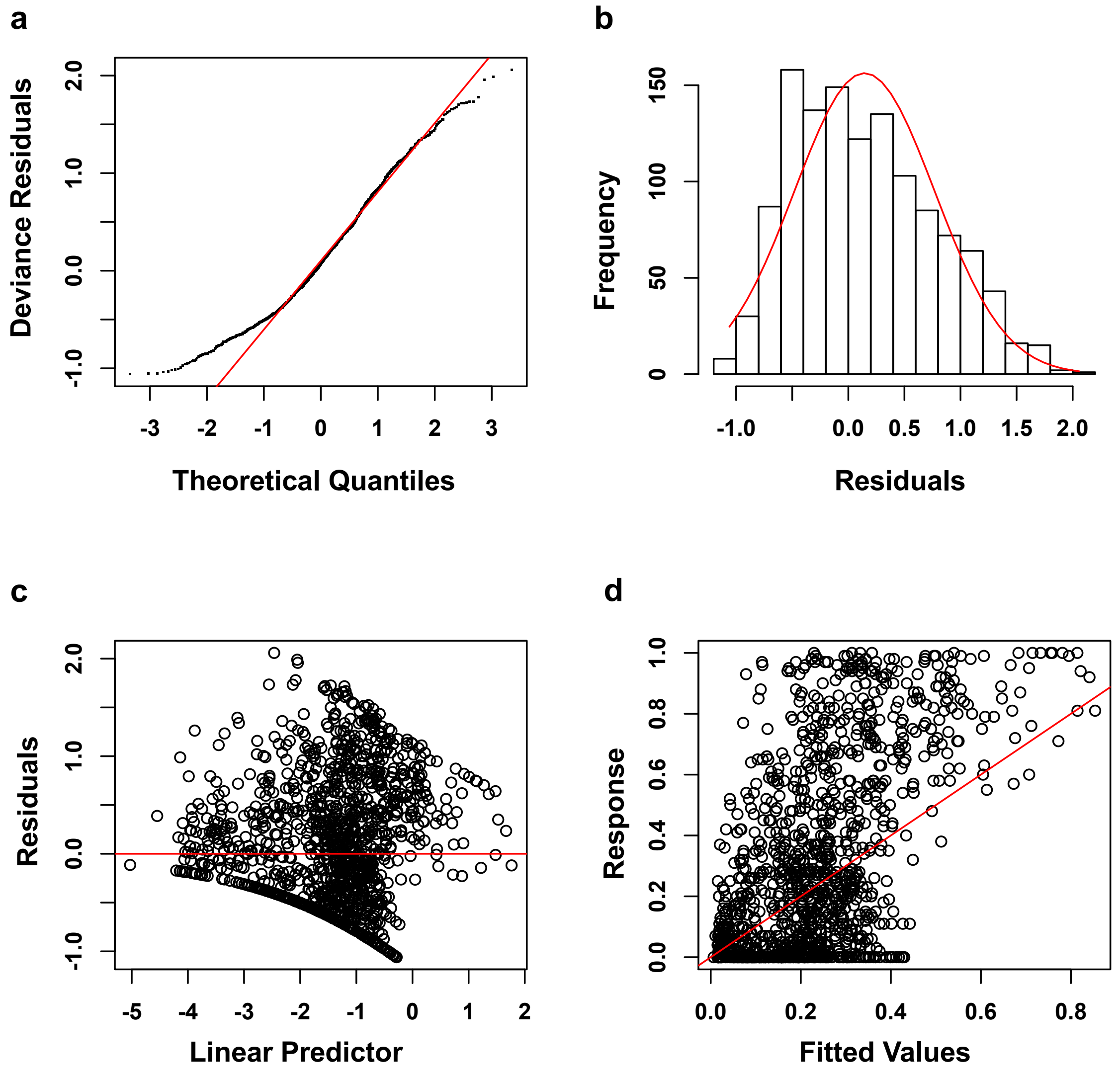

Figure S7. Diagnostic plots for final GAMM model on daily proportion of time spent ≥100 m by salmon sharks. (**a**) Quantile-quantile (Q-Q) plot for deviance residuals of the model with 1:1 line (red). (**b**) Distribution of normalized residuals with fitted normal curve (red). (**c**) Residuals versus linear predictor with zero-centred line (red). (**d**) Fitted values versus observed values with 1:1 line (red).

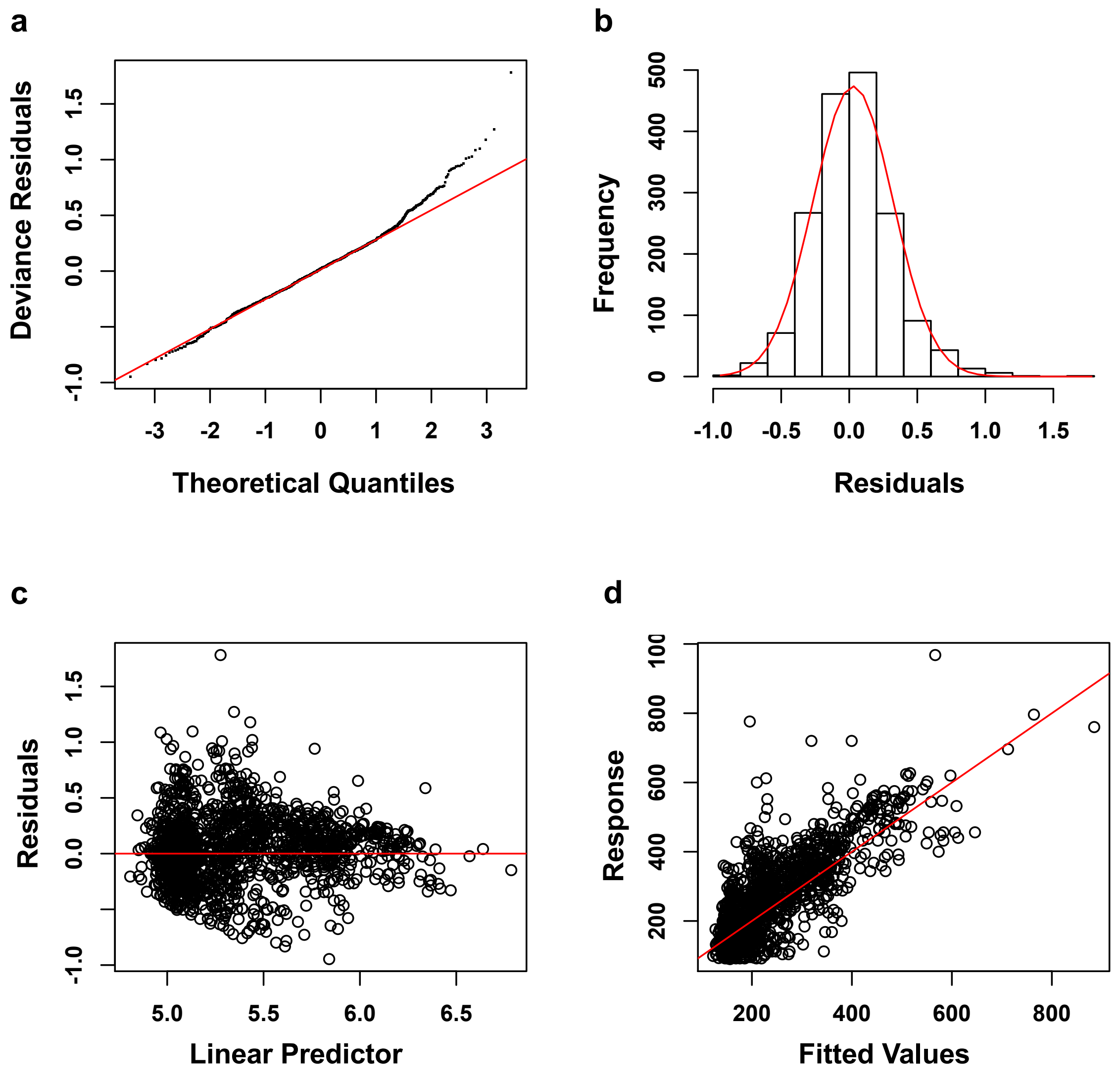

Figure S8. Diagnostic plots for final GAMM model on daily maximum depth of salmon sharks. (**a**) Quantile-quantile (Q-Q) plot for deviance residuals of the model with 1:1 line (red). (**b**) Distribution of normalized residuals with fitted normal curve (red). (**c**) Residuals versus linear predictor with zero-centred line (red). (**d**) Fitted values versus observed values with 1:1 line (red).

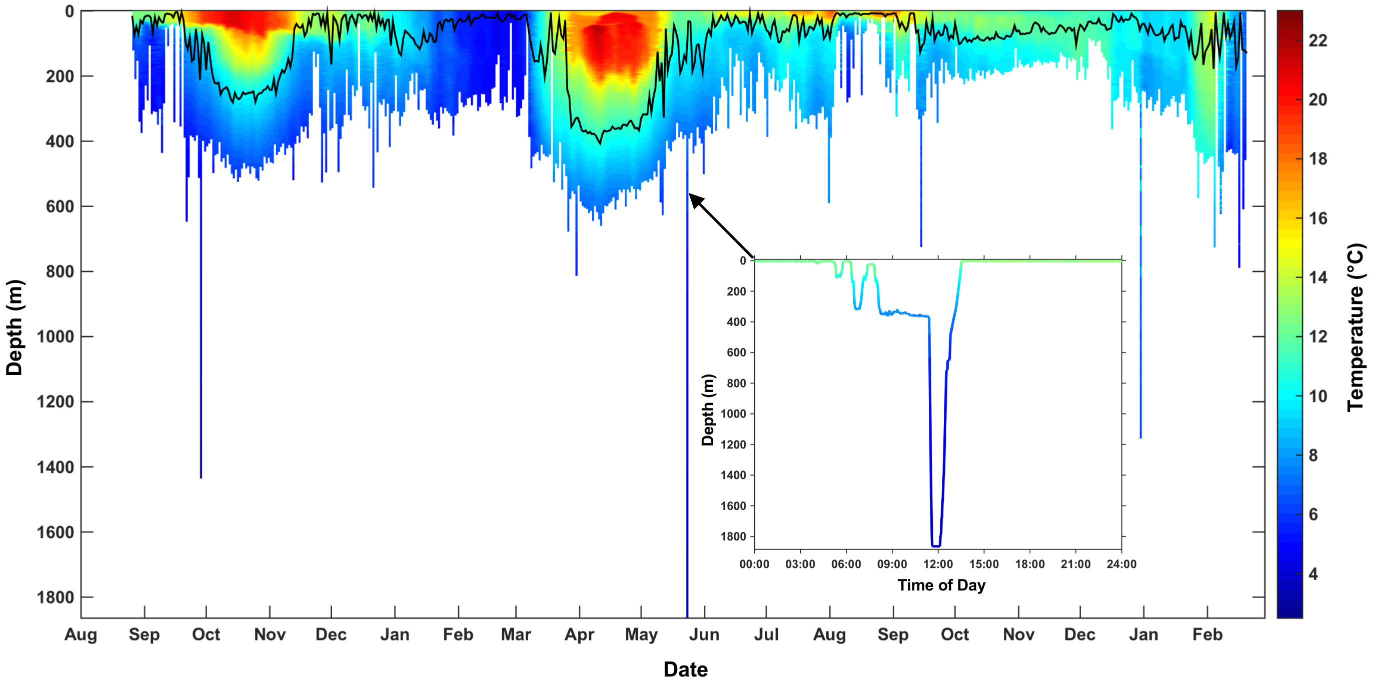

Figure S9. Water column thermal structure experienced by shark with implanted TDR-MK9 archival tag. Water column thermal structure is color-coded by temperature, and bottom limit denotes daily maximum depth. Solid line denotes daily overall mean depth. Ticks on x-axis denote the start of the month. Inset shows 24-h time series of depth (line) and temperature (colour) for the overall deepest dive profile. Note the pressure sensor maxed out at a depth of 1864 m.
